# STL-based Analysis of TRAIL-induced Apoptosis Challenges the Notion of Type I/Type II Cell Line Classification

**DOI:** 10.1371/journal.pcbi.1003056

**Published:** 2013-05-09

**Authors:** Szymon Stoma, Alexandre Donzé, François Bertaux, Oded Maler, Gregory Batt

**Affiliations:** 1 INRIA Paris-Rocquencourt, Le Chesnay, France; 2 VERIMAG, CNRS and the University of Grenoble, Gières, France; Princeton University, United States of America

## Abstract

Extrinsic apoptosis is a programmed cell death triggered by external ligands, such as the TNF-related apoptosis inducing ligand (TRAIL). Depending on the cell line, the specific molecular mechanisms leading to cell death may significantly differ. Precise characterization of these differences is crucial for understanding and exploiting extrinsic apoptosis. Cells show distinct behaviors on several aspects of apoptosis, including *(i)* the relative order of caspases activation, *(ii)* the necessity of mitochondria outer membrane permeabilization (MOMP) for effector caspase activation, and *(iii)* the survival of cell lines overexpressing Bcl2. These differences are attributed to the activation of one of two pathways, leading to classification of cell lines into two groups: type I and type II. In this work we challenge this type I/type II cell line classification. We encode the three aforementioned distinguishing behaviors in a formal language, called signal temporal logic (STL), and use it to extensively test the validity of a previously-proposed model of TRAIL-induced apoptosis with respect to experimental observations made on different cell lines. After having solved a few inconsistencies using STL-guided parameter search, we show that these three criteria do not define consistent cell line classifications in type I or type II, and suggest mutants that are predicted to exhibit ambivalent behaviors. In particular, this finding sheds light on the role of a feedback loop between caspases, and reconciliates two apparently-conflicting views regarding the importance of either upstream or downstream processes for cell-type determination. More generally, our work suggests that these three distinguishing behaviors should be merely considered as type I/II features rather than cell-type defining criteria. On the methodological side, this work illustrates the biological relevance of STL-diagrams, STL population data, and STL-guided parameter search implemented in the tool Breach. Such tools are well-adapted to the ever-increasing availability of heterogeneous knowledge on complex signal transduction pathways.

## Author Summary

Apoptosis, a major form of programmed cell death, plays a crucial role in shaping organs during development and controls homeostasis and tissue integrity throughout life. Defective apoptosis is often involved in cancer development and progression. Current understanding of externally triggered apoptosis is that death results from the activation of one out of two parallel signal transduction pathways. This leads to a classification of cell lines in two main types: type I and II. In the context of chemotherapy, understanding the cell-line-specific molecular mechanisms of apoptosis is important since this could guide drug usage. Biologists investigate the details of signal transduction pathways often at the single cell level and construct models to assess their current understanding. However, no systematic approach is employed to check the consistency of model predictions and experimental observations on various cell lines. Here we propose to use a formal specification language to encode the observed properties and a systematic approach to test whether model predictions are consistent with expected properties. Such property-guided model development and model revision approaches should guarantee an optimal use of the often heterogeneous experimental data.

## Introduction

Apoptosis, a major form of programmed cell death, plays a crucial role in shaping organs during development and controls homeostasis and tissue integrity throughout life [1], [2]. Moreover defective apoptosis is often involved in cancer development and progression [3]. Apoptosis can be triggered by *intrinsic* or *extrinsic* stimuli. Intrinsic apoptosis is triggered in case of cell damage (e.g. stress, UV radiation) or cell malfunction (e.g. oncogene activation). Extrinsic apoptosis is initiated by the presence of extracellular death ligands, such as Fas ligand (FasL), Tumor Necrosis Factor (TNF), or TRAIL [2]. Because the latter has a unique ability to trigger apoptosis in various cancer cell lines without significant toxicity toward normal cells, TRAIL-induced apoptosis has been the focus of extensive studies [1].

The effects of TRAIL application can be significantly different from one cell line to another [4]–[6]. The current understanding is that cell death results from the activation of one of two parallel pathways, leading to the classification of cell lines into two distinct cell types. In type I cells, effector caspases are directly activated by initiator caspases. Mitochondria outer membrane permeabilization (MOMP) is not required to generate lethal levels of caspase activity. In type II cells, the activation of initiator caspases triggers MOMP that in turn triggers effector caspases activation. MOMP is required for cell death. This necessity of mitochondrial pathway activation to undergo apoptosis is often referred as *type II phenotype*, in contrast to *type I phenotype* where MOMP is a side effect of apoptosis.

Many models of apoptosis, based on different mathematical formalisms, ranging from logical models to differential equation systems, have been proposed so far [2], [6]–[21]. To investigate the molecular origins of the two above-mentioned distinct phenotypes, Aldridge and colleagues developed a model describing key biochemical steps in TRAIL-induced apoptosis: extrinsic apoptosis reaction model (EARM1.4) [6]. EARM1.4 is an extension of a model developed to capture cell-to-cell variability in apopotosis of HeLa cells [15], [16]. In [6], the authors tested the hypothesis that the distinct cell behaviors can be explained solely by measured differences in protein concentrations before stimulation among different cell lines. Cell line models share the same set of ordinary differential equations and kinetic parameters, but possess specific protein contents at the initial state (i.e. before TRAIL application). These differences in the initial concentrations of a dozen of key apoptotic proteins are consistent with quantitative immunoblotting measurements. Then the authors use an abstract criterion that measures the influence of changes in initial protein concentrations on the future states of the system (i.e. divergence of trajectories): the direct finite-time Lyapunov exponent (DLE). They show that this criterion defines a partition of the state space that preserves known differences between phenotypes: type I and type II cells are associated to distinct regions in the state space [6]. The DLE-induced partition can be graphically represented as 2D slices of the high dimensional state space called DLE diagrams [6], [22]. As shown in [6], DLE diagrams are intuitive tools to predict the effect of mutations on cell type. However, the connection between the abstract DLE notion and cell phenotypes remains elusive: why type I and type II cells correspond to two different regions separated by a third one having high DLE values? Understanding this relationship is important to evaluate the general applicability of the proposed approach. Moreover in [6], the authors also probed the functioning of the apoptotic pathways in different cell lines and for different mutants using three different experimental methods: clonogenic assays, microscopy imaging and flow cytometry measurements of immunostained cells. These experiments probe subtly different aspects of the interplay of different pathway components, and most notably on the role of MOMP in the apoptotic response: death/survival following TRAIL stimulation of derived cell lines overexpressing Bcl2 (Property 1), synchronous/sequential activation of initiator and effector caspases (Property 2), and effector caspase activation prior/posterior to MOMP (Property 3). However, the authors do not test the consistency of EARM predictions with the detailed experimental information they provide.

**Figure 1:**
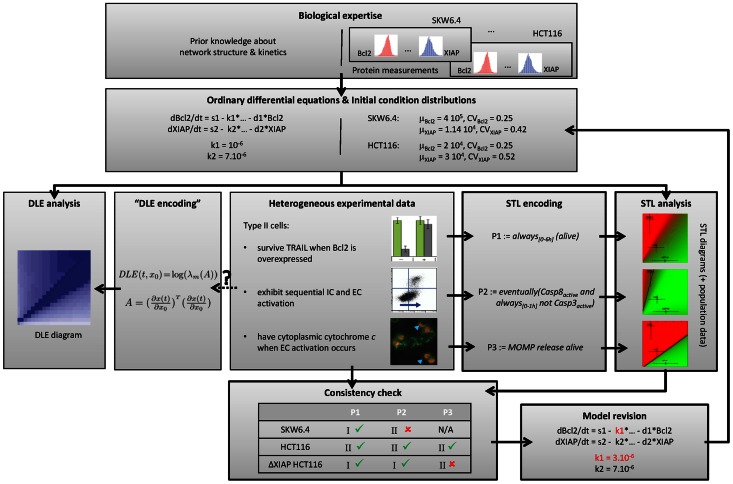
Property-based model analysis framework. Heterogeneous observations on the system are formalized as STL properties. Consistency between model and experimental observations is tested via STL diagrams and population data. Inconsistencies can be resolved via property-guided model revision. In contrast to DLE, STL properties explicitly encode specific aspects of cell's response, in our case, of the role of mitochondria in type I/II apoptosis. Bold boxes allows distinguishing our contribution with respect to the work of Aldridge and colleagues [6]. (Images reused with permission from Nature Publishing Group).

In this work we address the two above-mentioned problems by using a formal language, signal temporal logic (STL). STL was originally developed for monitoring purposes to specify the expected behavior of physical systems, including notably the order of physical events as well as the temporal distance between them [23]. Like other temporal logics and formal verification frameworks [24]–[31], it has been applied to the analysis of biomolecular networks [32], [33]. In particular, because it allows expressing in a rigorous manner transient behaviors of dynamical systems, one can encode as STL properties various cellular responses observed with different experimental methods and associated to type I/II phenotypes. Because STL properties have a quantitative interpretation, describing how robustly behaviors of the system satisfy or violate the property, STL diagrams can be constructed analogously to DLE diagrams. However, since STL diagrams are each associated to a specific STL property their interpretations do not suffer from ambiguities. Moreover, one can benefit from the expressive power of the STL language to encode detailed experimental information and thoroughly test the consistency of EARM with the various observations (Figure 1).

We report three findings. Firstly, our results highlighted that the three experimental methods proposed in [6] to investigate the importance of MOMP for cell death from three different perspectives, each suggesting a type I/II distinguishing criterion, do not lead to consistent cell line classifications. For example the ΔXIAP HCT116 cell line should be classified as type II based on Properties 1 and 2, and as type I based on Property 3. This challenges the well-posedness of the type I/II notion. Secondly, using our systematic approach, we found several inconsistencies between model predictions and actual observations. Taking again advantage of the quantitative interpretation of STL properties, we searched for valid parameters using a cost function that is minimal when all properties are consistent with experimental data and state-of-the-art global optimization tools. Inconsistencies have been resolved simply by modifying a few parameters, thus showing that there is no need for structural changes in the model. Thirdly, our findings reconciliate the apparently contradictory views expressed by Scaffidi and colleagues [5] and Aldridge and colleagues [6] about the origins of type I and II phenotypes. Indeed, Scaffidi, Barnhart and colleagues suggest that the initiator caspase activation capabilities are the main determinants of the type I/II phenotype of a cell line [5], [34], whereas Eissing and colleagues, Jost and colleagues, and Aldridge and colleagues suggest that the latter is mainly controlled by the relative abundance of downstream proteins, most notably XIAP and caspase-3 [4], [6], [7]. Our results suggest that, unlike downstream proteins, the modification of the concentration of upstream proteins within physiological range has a negligible effect on cellular responses. However, the critical effects of downstream protein concentration changes are fed back to upstream processes and are amplified via a positive feedback loop involving caspases 3, 6, and 8, leading to the activation of initiator caspases. Finally, the comparison of the STL and DLE diagrams showed that the DLE criterion essentially captures the notion of cell survival or cell death, like Property 1. This lead us to better understand why the fairly abstract DLE criterion induced biologically-relevant partitions in the work of Aldridge and colleagues [6]. A last contribution is that we extended the functionalities of the Breach tool [33] so that phase diagrams can be automatically computed given any differential equation model and STL property. Therefore, the methodology presented here can be applied to other complex biomolecular networks.

The first three sections of the Results part deal with the detailed analysis of three different observed phenotypes associated with type I/II behaviors, encoded in STL, and confronted with model predictions. In the last two sections, we study whether the EARM model can be reconciled with all the considered observations on all cell lines and search for the origins of cell type differences.

## Results

### Property 1: Type II cells survive if Bcl2 is over-expressed

#### STL encoding

**Figure 2:**
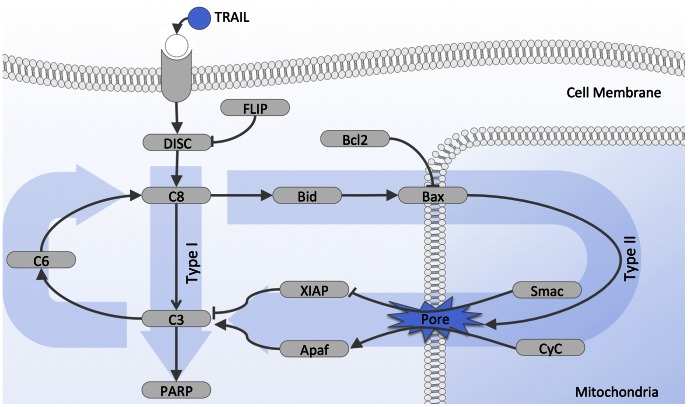
Simplified view on TRAIL-dependent apoptotic pathway. The activation of the membrane receptor by TRAIL binding promotes the assembly of the death-inducing signaling complexes (DISC), which recruit and activate initiator (pro-) caspases, including notably caspase-8 (C8) [53]. The recruitment of initiator caspases is modulated by FLIP. Once activated, initiator caspases cleave and activate effector caspases such as caspase-3 (C3). This effect is reinforced by a feedback loop involving caspase-6 (C6). Effector caspases cleave essential structural proteins, inhibitors of DNase, and DNA repair proteins (PARP), eventually leading to cell death. The cellular effect of effector caspase activation is regulated by factors such as the X-linked inhibitor of apoptosis protein (XIAP), which blocks the proteolytic activity of caspase-3 by binding tightly to its active site [54] and promotes its degradation via ubiquitination [55]. In addition to the direct activation of effector caspases, initiator caspases also activate Bid and Bax [56]. If not kept in check by inhibitors, most notably Bcl2, activated Bax directly contributes to the formation of pores in the mitochondria outer membrane, leading to MOMP [57]. Following MOMP, critical apoptosis regulators, such as Smac and cytochrome c (CyC), translocate into the cytoplasm. Smac binds to and inactivates XIAP, thus relieving the inhibition of effector caspases by XIAP [58]. Cytochrome c combines with Apaf-1 to form the apoptosome that in turn activates the initiator caspase-9 that activates effector caspases.

Bcl2 over-expression is the standard experimental method for distinguishing type I and type II cells [5]. Type I cells overexpressing the anti-apoptotic protein Bcl2 die in the presence of death ligand but type II cells survive. The sequestration of Bax by high levels of Bcl2 prevents the formation of pores in the mitochondrial outer membrane (Figure 2). Therefore clonogenic survival of an OE-Bcl2 derived cell line reveals the need for MOMP to trigger cell death in type II cells. Clonogenic survival data is available in [6] for three cell lines, SKW6.4 (human B lymphoma cells), HCT116 (human colon carcinoma cells), T47D (human breast carcinoma cells), and for the ΔXIAP mutant of HCT116 cells [6], [35], [36]. Cells were exposed to a 50 ng/ml TRAIL treatment for 6 hours.

Here, we encode in STL the observations made in clonogenic assays on HCT116, SKW6, and T47D cells [6]. Effector caspases cleave essential structural proteins and inhibitors of DNase, leading eventually to cell death. PARP is a substrate of these effector caspases and its cleavage is often regarded as a marker of commitment to death by cells [15], [16], [37]. Therefore, we consider here that a cell is alive if less than a half of the PARP proteins is cleaved. In STL, this translates into: *alive: = cPARP/PARP_total_<0.5*. Note that although the 50% threshold used here is somewhat arbitrary, we found that our conclusions are robust with respect to threshold changes in the range 10%–90% (see Figure S1). Then cell survival is simply expressed in STL as the cell is always alive: *Property1: = always_[0–6h]_(alive)*. Here, *always* is an STL keyword (see Methods). Its scope is limited to the first 6 hours as in experiments.

#### STL phase diagrams

**Figure 3:**
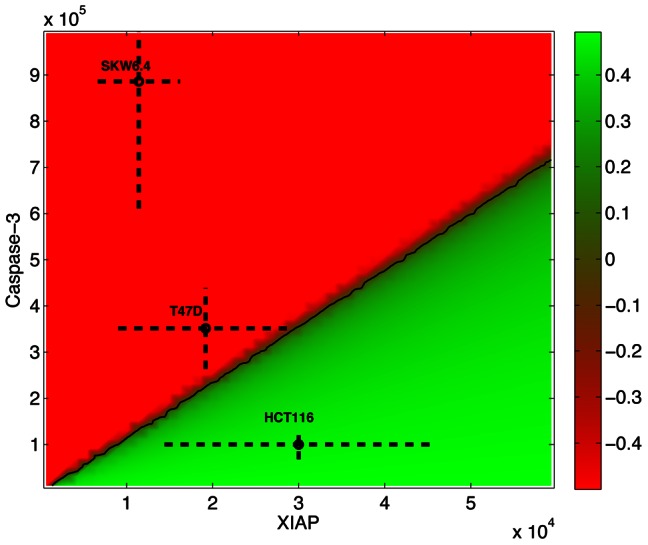
Property-1 phase diagram. Each point in the diagram represents a different initial concentration for XIAP and caspase-3 proteins, and its color represents the value of Property 1 evaluated on cell simulated behavior starting in these initial conditions (*p1: =  =  always_[0–6h]_(cPARP/PARP_total_<0.5*)). Other protein concentrations correspond to nominal protein concentrations for HCT116 cells (Table 1). Green regions satisfy the property (positive values). Red regions do not (negative values). Cell lines can be positioned in this diagram, using crosses which center and size are determined by the mean and standard deviation of measured protein concentrations (Methods and [6]).

For each initial protein concentration, one can predict the behavior of the system after TRAIL stimulation and assess whether this behavior satisfies a given STL property, or more precisely, estimate the value of the STL property given the behavior (see Methods). One can then graphically represent the value of the property in the state space by so-called phase diagrams (see Methods). The placement of cell lines in the phase diagram, based on their initial protein concentrations, indicates whether the cell line satisfies the given property (see Methods). Since it has been shown that the ratio of XIAP to caspase-3 concentrations plays a key role for the determination of the apoptotic type [6], we first constructed diagrams associated with these two variables. The corresponding STL phase diagram associated to Property 1 is represented in Figure 3. The death/survival property is tested in derived cell lines where Bcl2 is overexpressed (OE-Bcl2 cells; 10-fold increase of Bcl2 initial concentrations). The presence of two distinct regions in the diagram, one where Property 1 is satisfied (positive values, green) corresponding to cell survival, typical of type II cells, and one where Property 1 is falsified (negative values, red) corresponding to cell death, typical of type I cells, suggests that the model correctly predicts the importance of the XIAP/caspase-3 ratio as a key factor to determine cell survival following TRAIL treatment. We then positioned cell lines in the diagram based on measured mean and standard deviations of protein concentrations (see Methods). In agreement with the observations (Figure 2B in [6]) and the known type of these cell lines, the STL diagram predicts that OE-Bcl2 HCT116 cells do satisfy Property 1, but OE-Bcl2 SKW6.4 cells do not. OE-Bcl2 T47D cells are located close to separatrix and most cells satisfy Property 1. This is only in partial agreement with the fact that only half of T47D cells were found to survive (Figure 7C in [6]). Interestingly, as noted by Aldridge and colleagues, one can immediately see the consequences of mutations [6]. For example, ΔXIAP cell lines are shifted to the leftmost part of the diagram (regions with low XIAP concentrations) and are thus predicted to violate Property 1. That is, all OE-Bcl2/ΔXIAP mutants of the HCT116, SKW6.4, and T47D cell lines are predicted to die in clonogenic experiments. This is again in accordance with experimental observations for HCT116 cells (Figure 2B in [6]). A detailed comparison of the Property 1 diagrams and the DLE diagrams used in [6] shows that the successful classification of cells provided by DLE diagrams implicitly relies on the snap-action, all-or-none aspect of apoptosis (Figure S2). Using the approach we propose here, the property of interest is explicitly stated and the interpretation of the resulting diagrams is not ambiguous. Moreover, since STL is a property specification language, this framework can be applied to analyze other properties of the system, not necessarily relying on snap-action responses.

**Figure 7:**
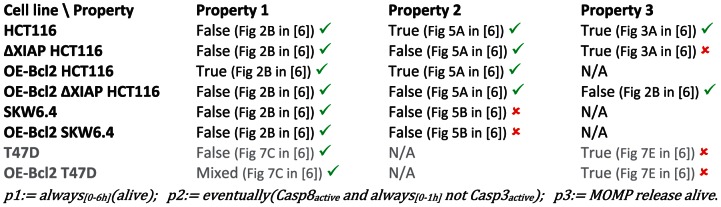
Summary of findings. Truth values of the three properties based on observations in [6] for the HCT116, SKW6.4, and T47D cell lines and some mutants. N/A indicates that the experimental information is not available. Experiments showed that OE-Bcl2 T47D cells present clonogenic survival rates close to 50%, hence their “mixed” behavior. Consistency or discrepancy with predictions from the original EARM 1.4 model obtained using our approach are indicated by green or red marks. Because of their ambiguous phenotypes, T47D cell data (in grey) were not used for parameter search.

#### STL population data

**Figure 4:**
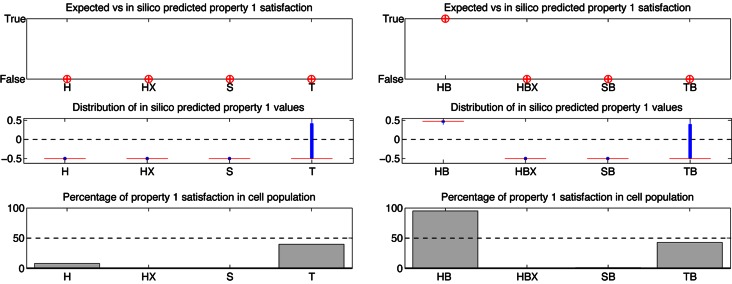
Property 1 population statistics. Plots indicate the satisfaction of Property 1 by the nominal cell (cross, top), the distribution of the values (middle), and the percentage of satisfaction (bottom) of Property 1 for populations of cells of different cell lines. For distributions, box boundaries and red line indicate first and third quartiles, and median, respectively. When experimental data is available, circles in the top plot represent the expected values. The following abbreviations are used in this and further figures: H is HCT116, HX is ΔXIAP HCT116, HB is OE-Bcl2 HCT116, HBX is OE-Bcl2/ΔXIAP HCT116, S is SKW6.4, SX is ΔXIAP SKW6.4, SB is OE-Bcl2 SKW6.4, SBX is OE-Bcl2/ΔXIAP SKW6.4, T is T47D, TX is ΔXIAP T47D, TB is OE-Bcl2 T47D and TBX is OE-Bcl2/ΔXIAP T47D.

Using STL diagrams helps in understanding how cell behavior depends on its initial protein content and hence suggests why cell lines exhibit different phenotypes. However, DLE and STL diagrams suffer from some limitations. In both cases, only two initial protein concentrations are modified (XIAP and caspase-3 in our examples). Therefore, they fail to capture all the differences between cell lines. In more precise terms, DLE and STL diagrams represent the value of the DLE or of an STL property in a 2D slice of the high-dimensional state space, and cell line distributions are projected onto the slice. Therefore, even if they provide insight into the behavior of cells that are affected by changes in initial protein concentrations, DLE and STL diagrams must be interpreted with care. The information is exact for the cell line used to construct the diagram, called the reference cell line, but it is only approximate for other, projected cell lines. To investigate how the diagrams change when reference cell line change, we constructed the Property 1 diagrams with respect to OE-Bcl2 HCT116, OE-Bcl2 SKW6.4, and OE-Bcl2 T47D cell lines (Figure S3A–C). Although the conclusions based on Figure 3 are indeed valid for HCT116 and SKW6.4 cell lines (OE-Bcl2 HCT116 cells survive, OE-Bcl2 SKW6.4 cells die), they differ for T47D cells. When using a slice of the state space based on OE-Bcl2 T47D cells, it appears that these cells are classified as exhibiting mixed-type behaviors (Figure S3C), as experimentally observed, instead of mostly type I as suggested by Figure 3. This example illustrates that problems may arise when placing different cell lines on the same phase diagram. To obtain a less comprehensive but more accurate view of the value of STL properties, we propose to use *STL population data* in combination with phase diagrams. Population data are statistics describing the STL property values associated to whole cell populations (see Methods). For Property 1, these statistics are presented in Figure 4 (and Figure S4 for all cell lines). One can first check that indeed the mean values, distributions and satisfaction rates of Property 1 are qualitatively consistent with the predictions we obtained from the STL diagram in Figure 3 for the OE-Bcl2 HCT116, OE-Bcl2 SKW6.4, OE-Bcl2 T47D, and OE-Bcl2/ΔXIAP HCT116 cells. Moreover, the satisfaction rates in Figure 4 can be directly compared with the experimentally-measured survival rates in clonogenic assays (Figure 2B and Figure 7C in [6]). Strikingly, our data shows excellent quantitative agreement with observed cell behaviors for all but the parental T47D cell line. Like in clonogenic assays, we predict the survival of a large majority of OE-Bcl2 HCT116 cells, half of the OE-Bcl2 T47D cells and a minority of HCT116 cells, the death of all ΔXIAP HCT116 and SKW6.4 cells and their OE-Bcl2 variants. The sole discrepancy concern T47D cells that are predicted to be more resistant to apoptosis than experimentally-observed.

### Property 2: Activations of initiator and effector caspases are sequential in Type II cells

#### STL encoding

In addition to survival of derived cell lines overexpressing Bcl2, Scaffidi and colleagues observed another important difference between type I and II cell lines: the dynamics of the activations of initiator and effector caspases by cleavage shows marked differences [5]. These are two critical events that can be considered as markers of the beginning and of the end of the apoptosis decision-making process. By using Western blots, Scaffidi and colleagues showed that in type I cells the activation of the effector caspase caspase-3 closely follows the activation of the initiator caspase caspase-8: caspase activations are gradual and near synchronous. In contrast, in type II cells the activation of initiator caspases is not closely followed by the activation of effector caspases [5]. Similar results have been obtained with a cellular resolution using FACS analysis (Figure 5 in [6]). The current understanding is that effector caspase activation is delayed until MOMP happens. Hence, the observed sequential activation is explained by a pre-MOMP delay in type II cells. Therefore this synchronous versus sequential activation is not only a robustly observed pattern but also relates to mechanistic interpretation of cell death.

**Figure 5:**
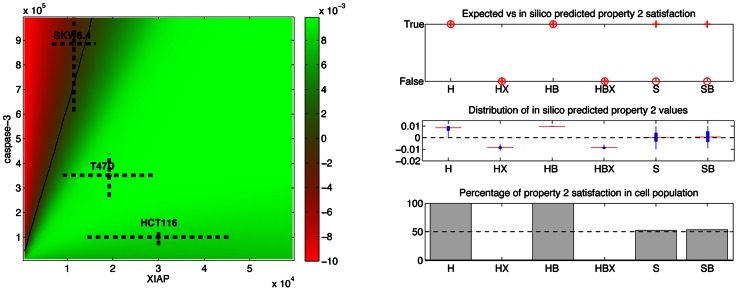
Property-2 phase diagram and statistics. Left: Values of Property 2 evaluated on cell simulated behaviors and represented as a function of the XIAP and caspase-3 initial concentrations (*p2: = eventually(Casp8_active_ and always_[0–1h]_ not Casp3_active_)*). Other protein concentrations correspond to nominal protein concentrations for HCT116 cells. As in Figure 3, cell lines are positioned in this diagram according to their protein initial concentrations. Right: distributions of the values of Property 2 across populations of cells of different cell lines. Notations are identical to those used in Figure 4. Note the discrepancy between the predicted (cross) and expected (circle) values for Property 2 in SKW6.4 cells.

We will consider that initiator and effector caspases activations are sequential if they are separated by more than one hour, in accordance with the low-temporal resolution of available observations in [5], [6]. So to express sequential activation, we say that “at some time point, caspase-8 is active and for at least an hour, caspase-3 remains inactive”. Hence, we have the following STL formula: *Property2: = eventually(Casp8_active_ and always_[0–1h]_ not Casp3_active_)*. We still have to set the threshold concentration for cleaved caspases that corresponds to a detectable activity. Since it has been shown that caspases are highly potent proteases (a few hundred caspases can cleave millions of substrate proteins within hours [38], [39]), we set this threshold concentration to 1% of the total caspase concentration: Casp8_active_: = Casp8*/Casp8_total_>1%, and Casp3_active_: = Casp3*/Casp3_total_>1% where Casp8* and Casp3* are the sum of the concentrations of all cleaved forms of caspase-8 and caspase-3, with the exclusion of caspase-8 bound to Bar and of caspase-3 bound to XIAP, respectively (the influence of the threshold is discussed in Figure S1).

#### STL phase diagrams and population data

Having formalized our property in STL, one can automatically construct the corresponding diagram (Figure 5, left). On this diagram one can clearly see two distinct, positive and negative, regions. HCT116 and T47D cells lie in the positive region and hence are predicted to satisfy Property 2, whereas SKW6.4 cells lie on the separatrix, and hence are predicted to show a mixed phenotype with respect to Property 2. Note that in the case of SKW6.4 cells, it is important to consider the diagram computed with respect to this cell line to have an accurate representation (compare S3D and E). The diagram also predicts that ΔXIAP mutants violate Property 2 (i.e. lie in the negative region). The predicted phenotypes of HCT116 and of ΔXIAP HCT116 cells are consistent with observations: whereas HCT116 cells show a clear sequential activation of caspases, this behavior is lost in ΔXIAP HCT116 cells [6]. Diagram shows that EARM1.4 is also compatible with the hypothesis that high levels of XIAP control caspase activation and substrate cleavage, and may promote apoptosis resistance and sublethal caspase activation *in vivo* [13]. However, the predicted phenotype of SKW6.4 cells is in contradiction with the observed one (Figure S3E and Figure 5 Right). As expected from type I cells, SKW6.4 cells clearly show synchronous activations of caspases and shoud therefore violate Property 2. The analysis of the OE-Bcl2 mutants of the HCT116, ΔXIAP HCT116, and SKW6.4 cell lines shows that consistent results are obtained in these cases (Figure 5, right). One should note that because caspase-3 is not activated in OE-Bcl2 HCT116 cells (they survive TRAIL treatment), Property 2 holds trivially in these cells. To investigate whether EARM1.4 can account for the observed phenotype of SKW6.4 cells, we slightly relaxed the timing constraint between the caspases activation times and found that by setting a slightly longer delay (e.g. 1h30 min), the mean value of Property 2 for the SKW6.4 cell population becomes negative as expected, and even more, that the percentage of cells satisfying Property 2 decreases to zero with longer delays. Therefore we conclude that the observed discrepancy results from EARM 1.4 limitations to capture quantitatively the elapse of time between events, rather than from severe modeling flaws.

### Property 3: MOMP precedes caspase-3 activation in Type II cells

#### STL encoding

In type I and type II cells, MOMP happens during apoptosis with comparable kinetics [5]. This is in apparent contradiction with the very different role of MOMP in the two pathways, as revealed by Bcl2 overexpression experiments (Property 1), and with the different kinetics of caspases activations (Property 2). The current understanding is that in type I cells MOMP is a consequence of effector caspases activation, whereas in type II cells, MOMP is the cause of effector caspases activation [5], [34], [40]. Under this assumption one should observe that in the first case MOMP follows effector caspases activation, and in the second case, that MOMP precedes effector caspases activation. This question has been directly investigated by Aldridge and colleagues by staining cells with anti-cytochrome c and anti-cPARP antibodies [6]. The authors demonstrate that most of HCT116 cells showing effector caspases activation also show cytoplasmic cytochrome c localization indicating that MOMP has happened. Stated differently, caspase-3 is not active until MOMP happens. This is not always true for ΔXIAP HCT116 cells, or for OE-Bcl2 ΔXIAP HCT116 cells: a significant proportion (respectively 20% and 80% of the cells) shows effector caspase activation in absence of cytoplasmic cytochrome c (Figure 3 in [6]). The same experiment was made for T47D and OE-Bcl2 T47D cells, showing that these cells behave like HCT116 cells: caspase-3 is not active until MOMP happens (Figure 7 in [6]).

To test the consistency of EARM1.4 with these observations, we express in STL the property, typical of type II behaviors, that cells remain alive until MOMP happens. We simply write *Property3: = MOMP release alive*. The *release* operator states that the second property (*alive*) must hold until the first property holds for the first time (*MOMP*). The occurrence of MOMP is detected by the titration of Apaf-1 by the released cytochrome c to form the apoptosome. We say that MOMP happened when more than 50% of Apaf-1 is bound to cytochrome c: *MOMP: = Apaf_free_/Apaf_total_<0.5* (see Figure S1 for discussion of threshold).

#### STL phase diagrams and population data

**Figure 6:**
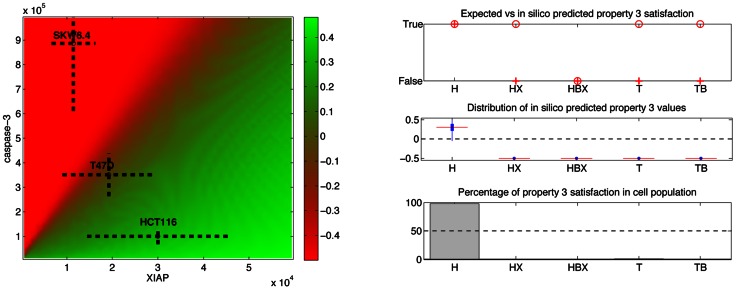
Property-3 phase diagram and statistics. The value of Property 3 (*p3: = MOMP release alive*) is represented as a function of the XIAP and caspase-3 initial concentrations. Other protein concentrations correspond to nominal protein concentrations for HCT116 cells. As in Figure 3, cell lines are positioned in this diagram according to their protein initial concentrations. Note that HCT116 cells depleted from all XIAP ([XIAP] = 0) are predicted not to satisfy Property 3. This is in contradiction to experimental observations [6]. (Right) Distributions of the values of Property 3 across populations of cells of different cell lines. Notations are identical to those used in Figure 4. One can note that discrepancies between predicted mean values (crosses) and observed phenotypes (circles) exist for ΔXIAP HCT116, T47D and OE-Bcl2 T47D cells.

We used Breach to compute the STL diagram associated with Property 3 with respect to HCT116 cells, and the Property 3 population data (Figure 6). One should note that like in the *in vitro* setup of [6], only cells in which MOMP happened were taken into account: we excluded surviving cells to compute statistics.

The diagram presented in Figure 6 is consistent with the observation that HCT116 cells satisfy the property. However, it suggests that the ΔXIAP mutant falsify the property, since negative values are found in the region where XIAP concentration is null. This is in contradiction with the observation that caspase-3 is active before MOMP happened in a majority (80%) of these cells. In summary, ΔXIAP HCT116 cells present a type I phenotype with respect to clonogenic survival and caspases activation dynamics, and a type II phenotype with respect to the need for MOMP for cell death, but the model classifies them as type I cells for all properties. In fact, the fact that ΔXIAP HCT116 cells have been observed to satisfy Property 3 but not Property 2 imposes strong constraints on the kinetics of the apoptosis process. In these cells, caspase-3 activation is precocious, since it follows by less than one hour the activation of caspase-8, implying that death (i.e. PARP cleavage) is rapid since it follows shortly after caspase-3 activation. But then Property 3 implies that MOMP happened even before this time instant. Given the efficient caspase-3 activation in EARM1.4, the model fails to capture the need for MOMP in these cells. Lastly, one can note that contradictions are also found with T47D, and OE-Bcl2 T47D cells (Figure 6, right).

### Improving EARM 1.4: Property-guided parameter search

In summary, we found that EARM1.4 satisfies the majority of the observed behaviors encoded in STL (Figure 7). This is commendable for a model of this size and complexity, given that EARM1.4 has not been tuned with respect to these properties, even if the model and the specific observations we used to state our STL properties have been published in the same paper [6]. However, few discrepancies were identified. It is important to test whether the proposed model is structurally not capable of accounting for all the observed properties. If not, this would call for significant model revision.

We first tried to resolve inconsistencies by minor adjustments of the thresholds we used in formulae. However, property satisfaction values proved robust to threshold changes (Figure S1). We therefore resorted to search for better parameter values using global optimization methods [41]. We defined a cost function that indicates for any given parameter how far the model is from satisfying all its constraints. More precisely, the cost function aggregates three measures: how many properties are consistent with the observations, how robustly satisfied or falsified they are, and how large are the deviations of the parameters with respect to their reference values. Then, we used the global optimization tool CMA-ES [42] to search automatically for parameters minimizing this cost function (see Methods). Here, one should note that the real-valued semantics of STL properties is critical: continuous optimization tools take advantage of the graded interpretation of STL properties, whose values indicate their “distances from satisfaction”. The sole use of traditional Boolean-valued interpretations of temporal logic formulae would have made this search impractical. Because of their ambiguous phenotypes, T47D cell data were not used for parameter search. We started with 43 parameters, that is, all catalytic and forward reaction rates (see Method section). After applying our optimization procedure we found that the modification of only 2 parameters was sufficient to achieve full agreement with experimental data. The parameters found by the search procedure are a parameter regulating the strength of the caspases feedback loop (2.71 fold increase) and a parameter regulating the kinetics of PARP cleavage (55.6 fold decrease) (Table S1). Given the usually large uncertainties in actual parameter values, such changes can still be considered as acceptable. New parameter values lead to satisfaction of Property 1–3 in *nominal* cells corresponding to all HCT116 and SKW6.4 normal and derived cell lines. To test whether property values are corrected at the *cell population* level, we recomputed the population data with these new parameter values. As shown in Figure S5, all inconsistencies were indeed resolved at the cell population level for all cell types (again with the exception of T47D cells).

### Origins of type I/II behaviors: Key role of downstream proteins and of a positive feedback loop

**Figure 8:**
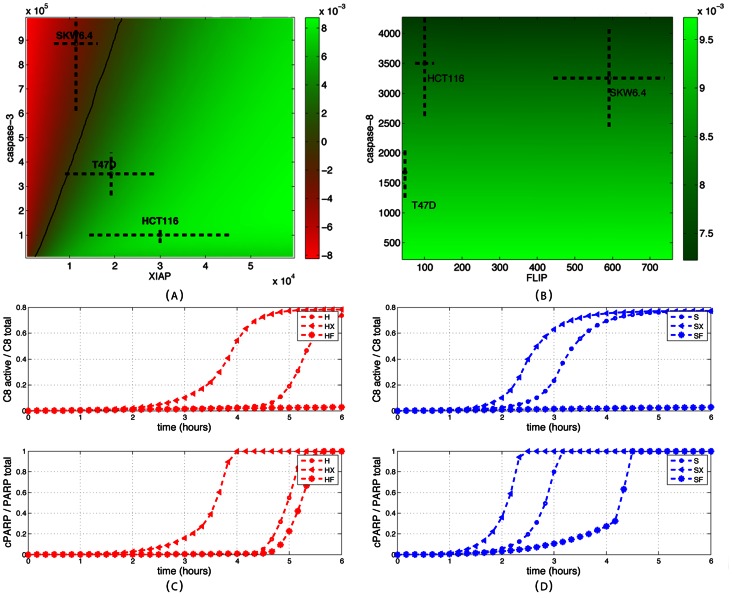
Investigating the role of upstream proteins. (A) XIAP/caspase-3 and (B) FLIP/caspase-8 Property 2 diagrams using HCT116 as reference cell line. Changes in XIAP/caspase-3 levels in HCT116 cells to match levels found in SKW6.4 cells change the original type II phenotype into a type I phenotype. This is not the case for FLIP/caspase-8 changes. (B) The corresponding DLE diagram does not offer intuitive interpretation. (C and D) Temporal evolution of active caspase-8 (top) and cleaved PARP (bottom) in HCT116 cells (red circle), SKW6.4 cells (blue circle), ΔXIAP HCT116 cells (red triangle), ΔXIAP SKW6.4 cells (blue triangle), ΔFB HCT116 (red star) and ΔFB SKW6.4 cells (blue star). The comparison of HCT116 and SKW6.4 cell lines with their ΔXIAP mutant shows important differences in the caspase-8 activation profile. Downstream proteins change upstream protein activation.

It is important to note that the significantly different phenotypic responses of the different cell lines are in the model solely explained by observed differences in the initial concentrations of a dozen of key proteins. Therefore one can use EARM1.4 with new parameter values (Table S1) and STL diagrams to investigate the *origins* of the different behaviors shown by cell lines. One important question is to distinguish whether the different behaviors can be explained exclusively by differences in upstream protein concentrations or exclusively by differences in downstream protein concentrations, or whether a combination of upstream and downstream changes is needed [6]. Indeed, it has been proposed that the main differences between type I and II behaviors are essentially due to differences in the efficiency of initiator caspase activation by the DISC [5], [34], [43]. It has also been proposed that the main determinant is the concentration of XIAP relative to caspase-3 [4], [6]. These questions can easily be answered using STL diagrams. Figure 8 shows the XIAP/caspase-3 and FLIP/caspase-8 diagrams for Property 2, computed with respect to the HCT116 cell line. It is apparent that the sole change of the concentrations of XIAP and capsase-3 from their original values to values corresponding to SKW6.4 cells is sufficient to alter the behavior of those cells from a type II to a type I phenotype. A similar change but for FLIP and caspase-8 proteins has no effect: cells remains with a type II phenotype. As illustrated in Figure S6 and S7, this is true for all properties and both directions (i.e., modifying protein concentrations from HCT116 to SKW6.4 values and vice-versa). This lack of influence of any upstream protein concentration is in apparent contradiction with the markedly different profiles for caspase-8 activation observed experimentally in [5] between type I and type II cells, and in EARM1.4 between HCT116 and SKW6.4 cell lines, and even more between normal and ΔXIAP cells that differ only in XIAP concentration (Figure 8C and 8D). The latter comparison suggests that differences in downstream protein concentrations feed back on upstream protein activities. To test this, we created feedback mutants (denoted ΔFB cells) by zeroing the cleaveage rate of caspase-8 by caspase-6. Similar activation profiles for caspase-8 in HCT116 and SKW6.4 cell lines were then obtained showing that in normal conditions differential activation of upstream processes is the consequence of differential downstream processes activation (Figure 8C and 8D). So, one can reconciliate the different views expressed by Scaffidi and colleagues and by Aldridge and colleagues [5], [6]. There are indeed functionally significant differences in upstream protein activities (e.g. caspase-8) in type I and II cells. However, according to EARM1.4 model, these differences do not result from differences in upstream protein levels but rather from downstream differences that feed their influence back on upstream processes. The feedback loop is required to preserve synchronous initiator and effector caspase activation in type I cells. Note that using STL was instrumental here. Indeed, because all cells die in these simulations (no Bcl2 overexpression), DLE diagrams were not offering relevant information.

Interestingly, the analysis of the FLIP/caspase-8 STL diagrams for Property 2 and 3 reveals that moderate inhibition of caspase-8 levels (e.g., by one third) in SKW6.4 cells would transform them into cells showing mixed type behaviors (Figure S7 D and F). Indeed the model predicts that these cells would present a sequential activation of caspases (Property 2 satisfied; a type II feature) and a MOMP-independent death (Property 3 violated; a type I feature). This mutant would show exactly the opposite behavior of ΔXIAP HCT116 cells, a combination of behaviors that has not yet been observed. Therefore, the detailed analysis of this cell line could possibly provide valuable information on the interplay between the two apoptotic pathways. Similarly, the partial inhibition of caspase-3 levels in SKW6.4 cells would also lead to cells showing mixed type behaviors (Property 1 remains false whereas Property 2 and 3 change to true; Figure S6 B, D, and F).

## Discussion

In this work, we expressed in a formal language, STL, a number of observed properties on molecular details of extrinsic apoptosis in several mammalian cell lines, and systematically tested their consistency with a previously-proposed model developed to capture the same process in the same cell lines, EARM1.4. It is important to note that even if model and experimental data have been published in the same article [6], the model has not been tuned to comply with the various observed properties we tested on the different cell lines. Indeed, we found several inconsistencies between model predictions and experimental observations. These inconsistencies can be resolved by model reparametrization involving a limited number of parameter changes. However, these needed changes were affecting key processes, namely the PARP cleavage rate and the strength of the caspases-3, -6 and -8 feedback loop. It is remarkable that the model was able to explain a number of experiments probing different aspect of apoptosis made on different cell lines and mutants, simply by taking into account observed differences in protein concentrations but keeping the same model structure and reaction rates for all cell lines. This makes it a valuable tool to investigate the *origins* of the two different cell responses. Unlike in *in vivo* experiments, the number of factors that could explain these differences is limited in EARM1.4. Using STL diagrams, we showed that observed differences in the concentrations of upstream proteins in different cell lines could not account for the observed cell type changes. This finding is consistent with the observation based on *in vivo* and *in silico* works that downstream proteins, most notably XIAP and caspase-3, play a key role [6], but is in apparent contradiction with the observation that upstream protein activities are markedly different in type I and II cell lines [5]. Detailed analysis showed that the effects of downstream protein concentration differences are in fact fed back to upstream processes and amplified via the positive feedback loop involving caspases 3, 6, and 8. This finding reconciliates the views expressed by Scaffidi and colleagues and by Aldridge and colleagues [5], [6].

Based on experimental observations, we defined three properties associated with type II behaviors: (*1*) the cell survives if Bcl2 is over-expressed, (*2*) the activations of initiator and effector caspases are sequential, and (*3*) MOMP precedes caspase-3 activation. They all assess the role of mitochondria for cell death and differ only in subtle means. However, they are not always equivalent. For example, ΔXIAP HCT116 cells satisfy Property 3 but not Property 2, leading to interpretations like ΔXIAP HCT116 being type I cells while exhibiting a type II phenotype. Based on our work, there is no evidence that one property could be considered as a defining criterion, excepted maybe for historical or practical reasons (cell types were originally defined based on caspase activation kinetics whereas Bcl2 overexpression is considered as the standard method for cell type classification). This challenges the consensual understanding that there exists (implicitly) well-defined type I and type II phenotypes. It should be noted that here we go beyond the notion of mixed cell type introduced by Aldridge and colleagues for describing T47D cells. The authors implicitly assume that cell types are well defined but that within a population of cells a mixture of both phenotypes can be observed, coming from cell-to-cell variability [16], [44], [45]. Here we propose that these three properties are considered as *type II features*. Then the ΔXIAP HCT116 cells would be more consistently qualified as possessing some type I and some type II features. With the accumulation of more detailed characterizations of apoptosis in more cell lines, it is likely that the use of the loosely-defined notion of cell types will otherwise become more and more problematic.

Like the DLE diagrams introduced by Aldridge and Haller [22], STL diagrams are a convenient and intuitive way to represent the influence of various factors on complex dynamical properties. However, STL diagrams are superior on several counts. Firstly, one can benefit from the expressive power of temporal logics to express different observed properties of the dynamics of the cell response. It allows us to test in which respect are the cell lines different. Secondly, although the evaluation of STL properties and of the DLE returns continuous values, the fact that STL values are signed – positive values indicate satisfaction and negative values indicate falsity – allows for a more direct interpretation of the diagrams. Moreover, it allows defining statistics over populations of cells. Thirdly, DLE generates well-defined partitions if in some regions a small change in the initial state has a mild effect on the future system's state, thus generating low DLE values, and in other regions, similar changes have drastic effects, thus generating high DLE values. Although this is clearly the case in cell lines overexpressing Bcl2 since some cells die, whereas others survive (Figure 3), this is not generally true.

DLE and STL diagrams are particularly useful to have a rapid view of the consequences of changing a few factors, initial concentrations in our case. This feature allows us to foresee the consequences of mutations (e.g. ΔXIAP mutants in XIAP/caspase-3 diagrams), to investigate the (lack of) influence of given factors (e.g. FLIP changes in FLIP/caspase-8 diagrams), and to assess the influence of cell-to-cell heterogeneity by representing graphically the means and standard values of populations in diagrams. However, heterogeneity in diagrams is limited to two dimensions. Moreover, since the cell lines differ in more than two dimensions, only one cell line can be correctly mapped in the state space slice of the diagram. Other cell lines are projected onto it, making their interpretations subject to caution. To solve this issue, we introduced population property values for describing the behavior of cell populations. These values and their statistics, notably means, standard deviations, and percentage of satisfaction, offer a more accurate view than phase diagrams. Indeed, even if we found that the rapid picture offered by STL diagrams are often consistent with population property values, a few cases illustrated the need to compute these statistics as well (e.g. T47D cells manifesting *in silico* a clear mixed-type behavior with respect to Property 1, that is not present in the phase diagram in Figure 3).

In addition to computing diagram and population statistics, STL properties also enable model revision based on *experimental observations*. Observed properties are encoded in STL and the continuous semantics of STL is used to search for valid parameter values. Traditional model revision methods based on curve fitting could not be adapted here by lack of well-defined time series data. The non-standard use of continuous semantics for temporal logic formula interpretation is essential to allow for an effective search [46]–[48]. Using global optimization methods, we found that the few discrepancies we had identified in earlier steps can be resolved by modifying only a restricted set of model parameters. Remarkably, one of the two selected parameters is regulating the strength of the caspases feedback loop, a process that is predicted to play an important role in the genesis of type I or type II behaviors.

The development of experimental methods to probe quantitatively subtle aspects of the dynamics of biological processes has spurred the development of large and complex quantitative models [49], [50]. However the available experimental data is seldom in the form of time series data directly usable by standard model validation and model calibration techniques. Therefore tools allowing for the exploration of model properties, the comparison between predictions and observations and the revision of models that are adapted to the available experimental data are increasingly needed. Temporal logics offer a flexible means to encode for a broad range of experimentally-observed properties. Moreover they are also formal languages that allow automating model analysis. Because it supports STL and uses by default distributions for parameter and initial concentrations, Breach naturally allows the exploration of properties of cell populations. We expect that Breach will become a valuable tool for the computational biologists to explore model properties, and more importantly, to get tight connections between experimental data and model predictions [51].

## Methods

### Modeling extrinsic apoptosis and cell line differences

We used the model of extrinsic apoptosis proposed by Aldridge and colleagues [6] named EARM1.4. This model is an extension and adaptation of a previous model, EARM1.0, proposed in [15]. EARM1.0 has been calibrated on HeLa cells using live and fixed cell imaging, flow cytometry of caspases substrates and biochemical analysis. EARM1.4 has been adapted to HCT116, SKW6.4 and T47D cells, and has been shown to capture their capacity to die or survive in OE-Bcl2 clonogenic experiments. It is a mass-action ODE model based on nearly 70 reactions and involving 17 native proteins, 40 modified proteins or protein complexes, and TRAIL. For each cell line, the model assumes different nominal initial protein concentrations. Nominal concentrations refer here to concentrations found in a hypothetical mean cell within the cell population. More precisely, out of the 17 native proteins, 12 have been quantified by immunoblotting and the relative differences between cell lines have been used to set nominal initial protein concentrations for HCT116, SKW6.4 and T47D cells (see Table 1). Besides initial concentrations, the 3 models are identical. ΔXIAP and OE-Bcl2 mutant cell lines are defined with respect to their parent cell line. In ΔXIAP cells, the XIAP concentration is set to 0. In OE-Bcl2 cells, the initial Bcl2 concentration is 10 times higher than in the parent cell line. For cells with modified feedback (ΔFB cells) we set the cleavage rate of caspase 8 by caspase 6, k7, to 0. To represent cell-to-cell variability within cell lines, we assumed that protein concentrations are log-normally distributed. The means of protein concentrations were the nominal values. The coefficient of variation were either measured, for caspase-3 and XIAP [6], or assumed to be 25% as in [16]. The complete model together with Breach is available in Supplementary Materials as MATLAB files (S9). The names of the variables, constants and reactions used in the model are the same as in [6].

**Table 1:** Initial concentrations of proteins in HCT116, SKW6.4, and T47D cells.

Protein\Cell line	HCT116	SKW6.4	T47D
**FLIP**	100 (0.25)	591 (0.25)	48 (0.25)
**caspase-8**	3500 (0.25)	3255 (0.25)	1680 (0.25)
**caspase-6**	10000 (0.25)	6700 (0.25)	22500 (0.25)
**caspase-3**	100000 (**0.32**)	886000 (**0.31**)	351000 (**0.25**)
**XIAP**	30000 (**0.52**)	11400 (**0.42**)	19200 (**0.53**)
**PARP**	1000000 (0.25)	1120000 (0.25)	1040000 (0.25)
**Bid**	40000 (0.25)	74800 (0.25)	53600 (0.25)
**Mcl1**	1000 (0.25)	1250 (0.25)	4640 (0.25)
**Bax**	80000 (0.25)	786400 (0.25)	113600 (0.25)
**Bcl2**	20000 (0.25)	400000 (0.25)	104000 (0.25)
**Smac**	100000 (0.25)	177000 (0.25)	139000 (0.25)

Nominal values and coefficients of variations for initial protein concentrations that differ between cell lines (see [6] for concentrations of other proteins). Protein concentrations are assumed log-normally distributed across the cell populations.

### STL semantics and property evaluation

**Figure 9:**
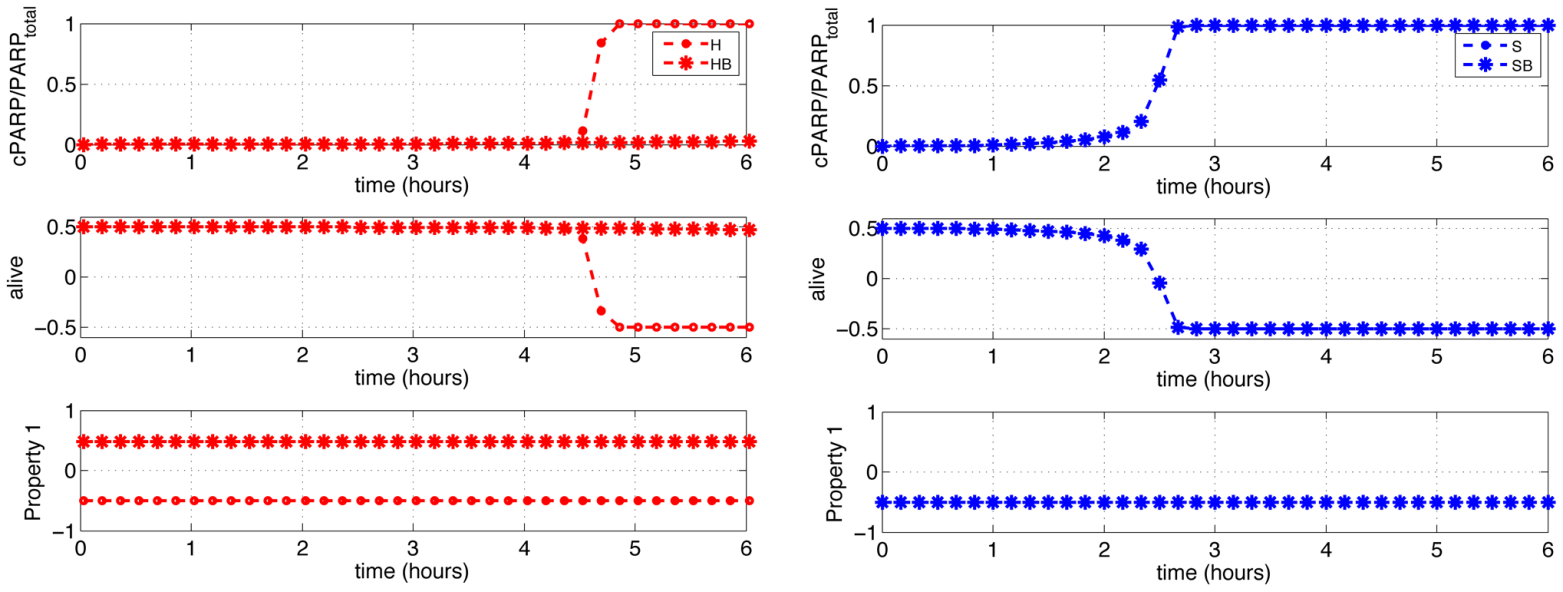
Evaluation of STL properties. Temporal evolution of the ratio *cPARP/PARP_total_*, of the value of the property *alive*: = *cPARP/PARP_total_<0.5*, and of *Property 1*: = *always_[0–6h]_( cPARP/PARP_total_<0.5*) for HCT116 and OE-Bcl2 HCT116 cells (left), and SKW6.4 and OE-Bcl2 SKW6.4 cells (right). When the concentration of cleaved PARP increases, the value of the *alive* property gradually decreases from a positive value (“true”) to a negative value (“false”). *Property 1* at time *t* evaluates to the minimal value of *alive* at all future times. So, Property 1 simply captures whether at all times *alive* holds.

STL is an intuitive yet formal language for specifying the properties of continuous dynamical systems. It allows us to express in a (pseudo-) natural language hypothesis on the mechanistic functioning of the system taken from available biological knowledge in a formal way so that model consistency can be precisely and systematically tested. Given a model of the system, expected properties are expressed using predicates describing constraints on protein concentrations, like *cPARP<10^5^*, traditional logical operators, like *and, or* and *implies*, and temporal operators, like *eventually_[a,b]_, always_[a,b]_*, and *until_[a,b]_*. Time intervals [a,b] limit the scope of temporal operators. These operators can be combined to create properties of arbitrary complexities. For example, *always_[0–6h] _(XIAP>10^3^ and cPARP<10^5^)* is a valid STL formula. The formal syntax is given in Table S2 (top). STL properties are then interpreted with respect to so-called signals. In this context, signals are functions from time to the reals representing the evolution of the different concentrations in the system. Computationaly speaking, they often come from (discrete) time-series data obtained by numerical simulation of the ODE model. The semantics is defined such that it captures a notion of distance from satisfaction. For example, the interpretation at time *t* of the predicate *XIAP>10^3^* is simply the value of *XIAP(t) - 10^3^*. Trivially it is positive if *XIAP>10^3^*, and negative if *XIAP<10^3^*. The interpretation of *XIAP>10^3^ and cPARP<10^5^* at time *t* is the minimum of the value of the two operands at time *t*. Note that it is positive if and only if both operands have positive values. Similarly, the interpretation of *always(XIAP>10^3^)* is the minimum of the value of *XIAP>10^3^* at all future time instants. It is positive if *XIAP* is always greater than *10^3^*. The interpretation of STL formulas is also illustrated on Figure 9. More generally, the continuous interpretation of STL properties ensures that if the value of a property is positive (resp. negative), then the property holds (resp. is violated) in a more usual Boolean interpretation. Moreover, it captures a notion of “distance from satisfaction”: a large positive value indicates a robustly satisfied property, whereas a large negative value indicates a property that is far from satisfaction. The semantics is formally defined in Table S2 (bottom). Note that property values are relative to the formula, in the sense that values obtained for different STL formulas are not directly comparable between each other.

### Computation of property diagrams

Given an STL property, the associated STL phase diagram is a representation of the value of the property as a function of the system's initial configuration. More precisely diagrams represent property values in 2D slices of a high dimensional state space. Each point in the diagram is associated to the value of the STL formula evaluated on the system's trajectory starting at this point. Boundaries were set so as to enclose the variability observed between cell lines. Diagrams are defined with respect to a particular cell line: with the exception of the two variables of the diagram, all other variables assume their nominal values for the given cell line. Other cell lines are placed on the diagram based on the initial concentrations of the selected proteins. Unless mentioned otherwise, the HCT116 cell line is used as a reference. In practice, a 50×50 grid of linearly-spaced points is used for the computation of each diagram. For each point on the grid, we computed the cell behavior predicted by the model and then the value of the STL property associated with this behavior (see Program S1). The ranges for caspase-3, XIAP, caspase-8 and FLIP are [0, 10^6^], [0, 6*10^4^], [1200, 4500] and [0, 800], respectively. Similarly, DLE diagrams represent the direct finite-time Lyapunov exponent for all points in (a 2D slice of) the state space. This value captures the sensitivity to initial conditions: 
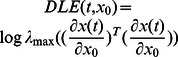
, where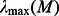 denotes the maximum eigenvalue of the matrix *M* and *t* is some future time instant (here 6 or 4 hours). Again, a 50×50 grid of linearly-spaced points is used for the computation of DLE diagrams.

### Computation of STL population data

Given an STL property, the STL population data correspond to the evaluation of this property on all the simulated individual cell behaviors among a population of cells of a given cell line. Based on these property values, statistics are computed. For STL population data, 5000 different initial conditions are obtained for each cell line by sampling around its nominal initial conditions from lognormal distributions. Mean values, value distributions and percentages of satisfaction of the property (i.e. the percentage of cells in the population satisfying a given property) are then computed.

### Parameter search procedure

The search procedure has two phases. In the first phase we search for new parameters for EARM1.4 that lead to full agreement with experimental data (Figure 7). In the second phase, when a solution is found, we minimize the number of modified parameters. We use a cost function composed of three different components: continuous, Boolean, and parameter penalties. The continuous penalties correspond to the (negation of) the continuous values of STL properties, and the Boolean penalties correspond to their Boolean value multiplied by a (negative) constant. These costs decrease when more properties are consistent with observations (B_penalty_), and when they are more robustly consistent with observations (C_penalty_). In the continuous component, weights are used to balance the importance of all properties, given their typical range. The last component penalizes parameter deviations from their original values (P_penalty_). The overall cost is the weighted sum of these three components.
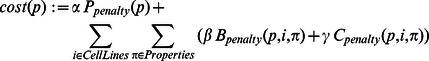
In the first phase, we selected 43 parameters (14 catalytic rates of enzymatic reactions and 29 forward rates) out of approximately 80 parameters in EARM1.4. Parameter modifications were limited to a 100-fold change. We set weights so that the Boolean, continuous, and parameter penalties contributed to approximately 50%, 30%, and 20% of the cost, respectively. After 10 hours of computations (2.2 GHz processor, 8GB RAM), the search converged to a state in which all expected properties were satisfied by the model (T47D cells excluded).

In the second phase, we selected the parameters that changed by more than 5 folds (there were 5 such parameters: kc9, kc25, kc20, k7 and k24) and run the search again for each pair of these parameters. The cost function was modified by setting the C_penalty_ parameter to 0, and the beta parameter such as the Boolean penalty was responsible for approximately 90% of the cost. As a result, parameter deviations were minimized while preserving the agreement with the experimental data. We found that reparametrization of only one pair of parameters allowed for satisfaction of all properties for all cell lines.

### Breach tool

All the computations have been made using Breach [33], [48]. This MATLAB/C++ toolbox allows for efficient numerical simulation, for sensitivity computation, and for STL property and DLE evaluation. In particular, DLEs can efficiently be computed via forward sensitivity analysis [52]. Breach is particularly oriented towards the analysis of parametric systems, in the sense that it offers efficient routines for global sensitivity analysis and parameter search, and that the graphical user interface facilitates the modification of parameters and initial conditions, and the exploration of parameter spaces.

## Supporting Information

Figure S1 **Formula robustness.** Number of matches between predicted and observed satisfaction values for Properties 1–3 in all HCT116 and SKW6.4 cell lines (Figure 7) as a function of the PARP-related threshold, α, defining the *alive* property, of the Apaf-related threshold, β, defining the *MOMP* occurrence and of the caspase-related threshold, γ, defining caspases activation when (A) α and β vary, and γ is fixed, or (B) γ varies, and α and β are fixed. Thresholds α, β, and γ *are defined as follows: p1: = always_[0–6h]_(cPARP/PARP_total_<*α); *p2: = eventually(Casp8_active_ and always_[0–1h]_ not Casp3_active_); p3: = Apaf_free_/Apaf_total_<*β *release (cPARP/PARP_total_<*α)), where Casp8_active_: = Casp8*/Casp8_total_>γ and Casp3_active_: = Casp3*/Casp3_total_>γ. Full consistency with all experimental data corresponds to 16 matches (15 in Figure 7 and, additionally, p2(SKW6.4) = True). For original properties (α = β = 50% and γ = 1%), we found three mismatches (Figure 7). This number is robust with respect to changes of the PARP-related threshold, α, and of the Apaf-related threshold, β. It is also robust to the caspase-related threshold, γ, provided that this value remains low enough (i.e. <2%).
(TIF)

Figure S2 **Comparison between DLE and Property 1 STL diagrams.** Diagrams representing the values of the DLE computed at time T (A,C) and of the STL Property: = *always_[0-T]_(cPARP/PARP_total_<*0.5) (B,D) for T = 6 h (A–B) and T = 4 h (C–D). Strikingly, for the two time instants the separatrix is exactly at the same position, revealing that DLE and Property 1 capture precisely the same behavior: the existence of two different possible outcomes: survival or death. However, in full generality the DLE simply measures the influence of small changes in initial protein concentrations on the future state of the system. In fact, this similarity comes from the *snap-action* aspect of apoptotic cell death, captured by the EARM model: cell death is immediately preceded by a sudden activation of effector caspases (all-or-none behavior) [15]. Small changes in initial protein concentrations will result in dramatic differences in protein concentrations at the time of death and therefore in high DLE values. One should also note that the interpretation of low DLE values is ambiguous, since low values are found in regions corresponding to type I (SKW6.4) and to type II cell types (HCT116).
(TIF)

Figure S3 **XIAP/capsase-3 STL diagrams for all properties and using HCT116, SKW6.4 or T47D as reference cell line.** Diagrams representing the values of the STL properties p1 (A–C), p2 (D–F) and p3 (G–H) computed using HCT116 (A,D,G), SKW6.4 (B,E,H), or T47D (C,F,I) nominal protein concentrations. Bcl2 is overexpressed in Property 1 diagrams. In most cases, for a given property the satisfaction values associated with each cell type is similar irrespectively of the reference cell line used to construct the diagram. However, there are exceptions, like in the case of T47D cell line behavior (H and I). So care must be taken when interpreting STL diagrams. The same situation holds with DLE diagrams (not shown).
(TIF)

Figure S4 **STL property values across all cell lines for Properties 1–3 for the EARM1.4.** For each property, plots indicate the nominal cell value (top), the distribution (middle), and the percentage of satisfaction (bottom) of the property values for populations of cells of different cell lines. Notations are identical to those used in Figure 4.
(TIF)

Figure S5 **Population statistics for Property 1, 2 and 3, computed with new parameter values.** (see Table S1) This data should be compared with Figure 4, 5 (right), and 6 (right). The new parameter values allow resolving the inconsistencies found for SKW6.4, OEBcl2 SKW6.4 cells for Property 2, and for ΔXIAP HCT116 cells for Property 3. T47D cells still do not satisfy Property 3 as expected. Notations are identical to those used in Figure 4.
(TIF)

Figure S6 **XIAP/Capsase-3 STL diagrams computed with new parameter values for all properties and using HCT116 or SKW6.4 as reference cell lines.** Diagrams representing the values of the STL properties p1 (A–B), p2 (C–D) and p3 (E–F), computed using HCT116 (A,C,E) or SKW6.4 (B,D,F) nominal protein concentrations.
(TIF)

Figure S7 **FLIP/Capsase-8 STL diagrams computed with new parameter values for all properties and using HCT116 or SKW6.4 as reference cell lines.** Diagrams representing the values of the STL properties p1 (A–B), p2 (C–D) and p3 (E–F), computed using HCT116 (A,C,E) or SKW6.4 (B,D,F) nominal protein concentrations.
(TIF)

Program S1 **Computation of STL diagrams using Breach** [**33**] **.** The archive contains the freely-distributed Matlab tool Breach, an implementation of EARM1.4 in Breach, initial conditions for each of 12 cell lines used in this article, and example scripts illustrating how to generate STL phase diagrams.
(ZIP)

Table S1 **Valid parameters.** List of minimal parameter set leading to Property1–3 satisfaction for all but T47D cells, together with their new and original values, and the corresponding fold change.
(TIF)

Table S2
**Syntax and semantics of STL** [**48**] **.** The syntax of STL formulas is defined inductively. Here, 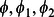 are STL formulas, 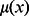 is an equality of type 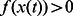, with *f* a real-valued function on the state *x*, and *[a,b]* is a time interval. The real-valued semantics 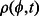 of an STL formula φ at time *t* is interpreted on a real-valued signal *x(t)* defined on a time interval *[0,T_f_]*, where *T_f_* is typically the end time of a simulation. One additionally defines 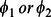 as 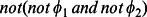, 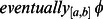 as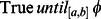 and 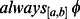 as 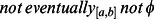.(TIF)
