# Supplementary figures and images for: STL-based Analysis of TRAIL-induced Apoptosis Challenges the Notion of Type I/Type II Cell Line Classification
Source: PLoS Comput Biol. 2013 May 9;9(5):e1003056. doi: 10.1371/journal.pcbi.1003056 (PMC3649977; doi:10.1371/journal.pcbi.1003056)

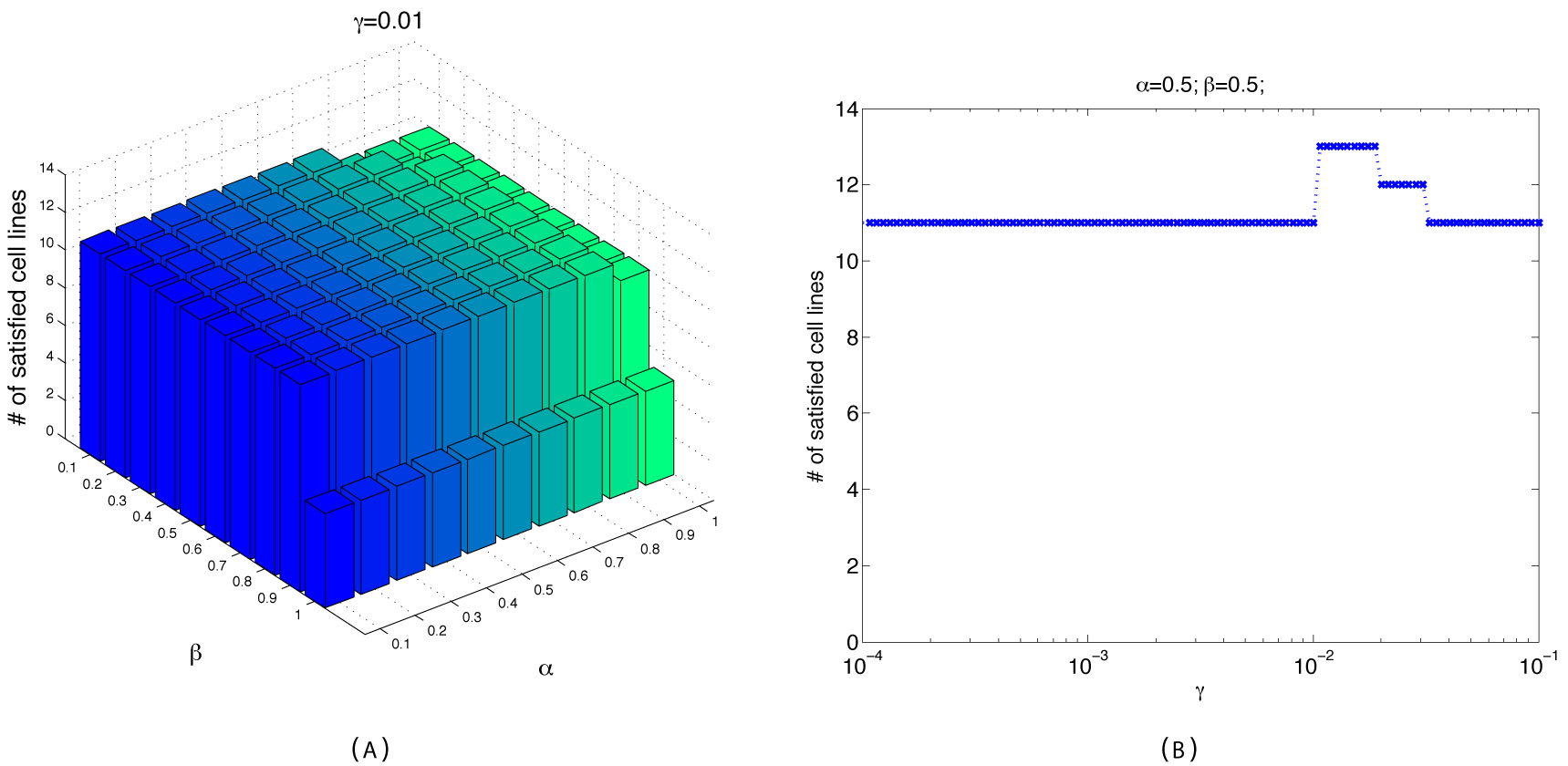

Supplement: Figure S1 — Formula robustness. Number of matches between predicted and observed satisfaction values for Properties 1–3 in all HCT116 and SKW6.4 cell lines (Figure 7) as a function of the PARP-related threshold, α, defining the alive property, of the Apaf-related threshold, β, defining the MOMP occurrence and of the caspase-related threshold, γ, defining caspases activation when (A) α and β vary, and γ is fixed, or (B) γ varies, and α and β are fixed. Thresholds α, β, and γ are defined as follows: p1: = always[0–6h](cPARP/PARPtotal<α); p2: = eventually(Casp8active and always[0–1h] not Casp3active); p3: = Apaffree/Apaftotal<β release (cPARP/PARPtotal<α)), where Casp8active: = Casp8*/Casp8total>γ and Casp3active: = Casp3*/Casp3total>γ. Full consistency with all experimental data corresponds to 16 matches (15 in Figure 7 and, additionally, p2(SKW6.4) = True). For original properties (α = β = 50% and γ = 1%), we found three mismatches (Figure 7). This number is robust with respect to changes of the PARP-related threshold, α, and of the Apaf-related threshold, β. It is also robust to the caspase-related threshold, γ, provided that this value remains low enough (i.e. <2%). (TIF) [file pcbi.1003056.s001.tif]

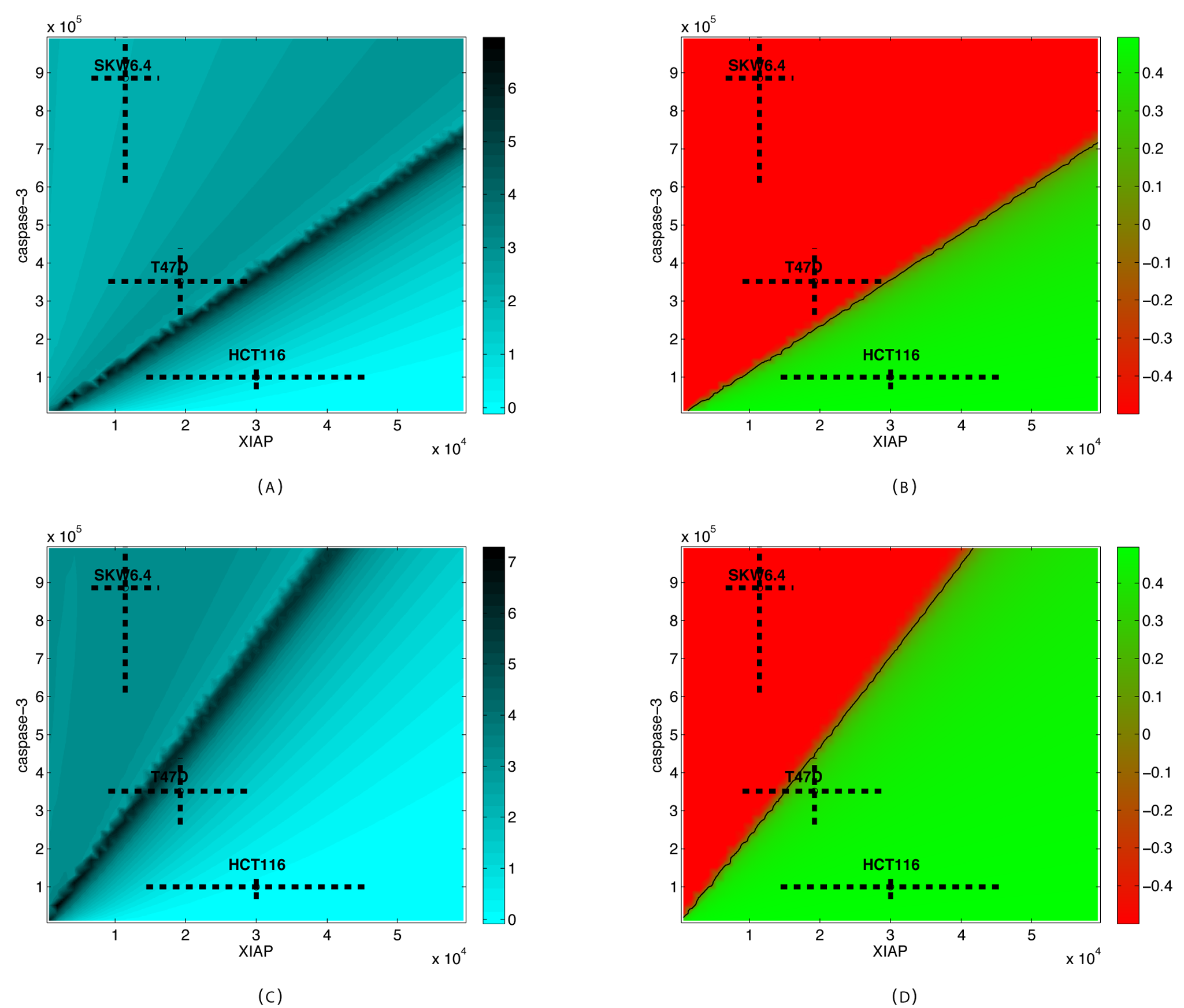

Supplement: Figure S2 — Comparison between DLE and Property 1 STL diagrams. Diagrams representing the values of the DLE computed at time T (A,C) and of the STL Property: = always[0-T](cPARP/PARPtotal<0.5) (B,D) for T = 6 h (A–B) and T = 4 h (C–D). Strikingly, for the two time instants the separatrix is exactly at the same position, revealing that DLE and Property 1 capture precisely the same behavior: the existence of two different possible outcomes: survival or death. However, in full generality the DLE simply measures the influence of small changes in initial protein concentrations on the future state of the system. In fact, this similarity comes from the snap-action aspect of apoptotic cell death, captured by the EARM model: cell death is immediately preceded by a sudden activation of effector caspases (all-or-none behavior) [15]. Small changes in initial protein concentrations will result in dramatic differences in protein concentrations at the time of death and therefore in high DLE values. One should also note that the interpretation of low DLE values is ambiguous, since low values are found in regions corresponding to type I (SKW6.4) and to type II cell types (HCT116). (TIF) [file pcbi.1003056.s002.tif]

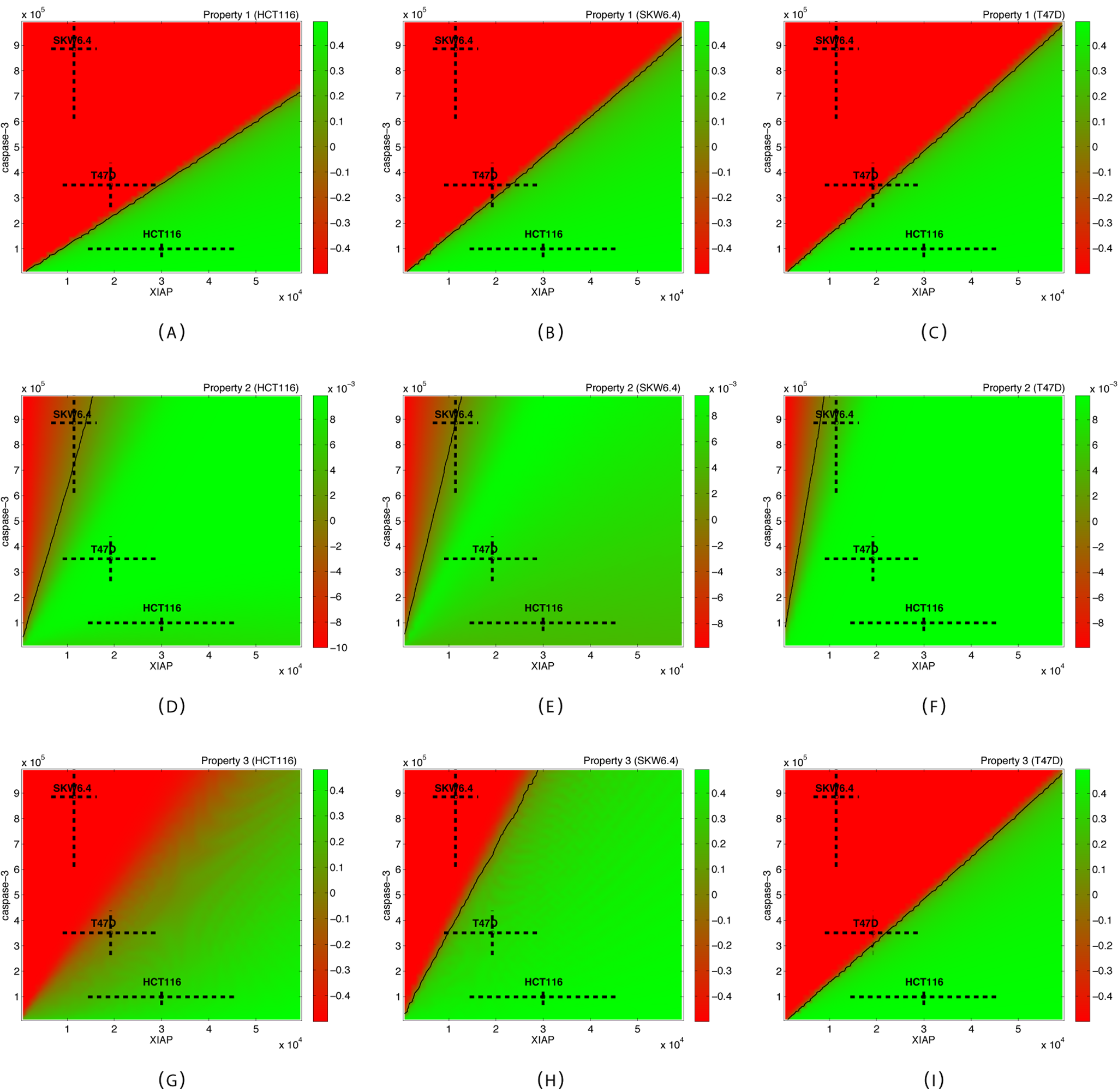

Supplement: Figure S3 — XIAP/capsase-3 STL diagrams for all properties and using HCT116, SKW6.4 or T47D as reference cell line. Diagrams representing the values of the STL properties p1 (A–C), p2 (D–F) and p3 (G–H) computed using HCT116 (A,D,G), SKW6.4 (B,E,H), or T47D (C,F,I) nominal protein concentrations. Bcl2 is overexpressed in Property 1 diagrams. In most cases, for a given property the satisfaction values associated with each cell type is similar irrespectively of the reference cell line used to construct the diagram. However, there are exceptions, like in the case of T47D cell line behavior (H and I). So care must be taken when interpreting STL diagrams. The same situation holds with DLE diagrams (not shown). (TIF) [file pcbi.1003056.s003.tif]

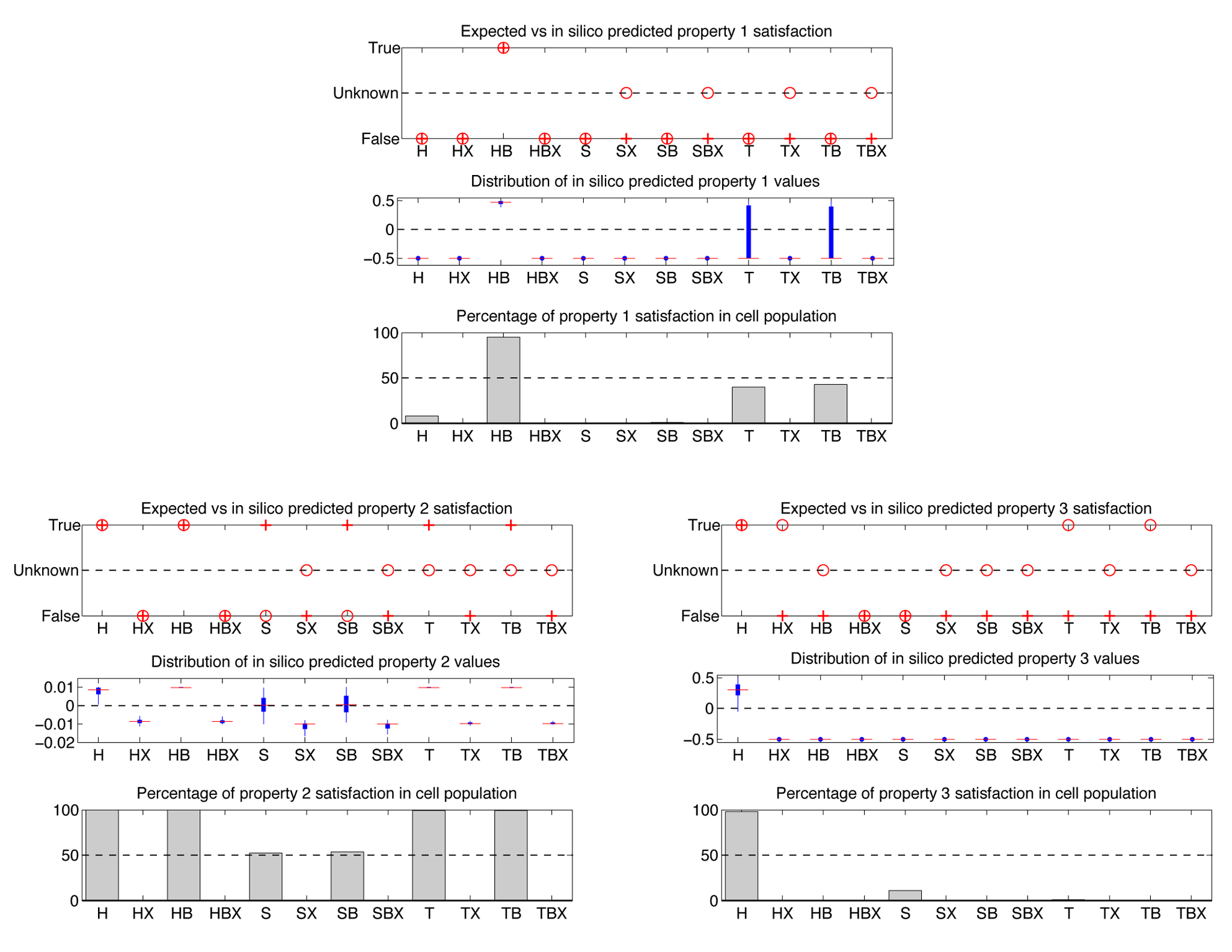

Supplement: Figure S4 — STL property values across all cell lines for Properties 1–3 for the EARM1.4. For each property, plots indicate the nominal cell value (top), the distribution (middle), and the percentage of satisfaction (bottom) of the property values for populations of cells of different cell lines. Notations are identical to those used in Figure 4. (TIF) [file pcbi.1003056.s004.tif]

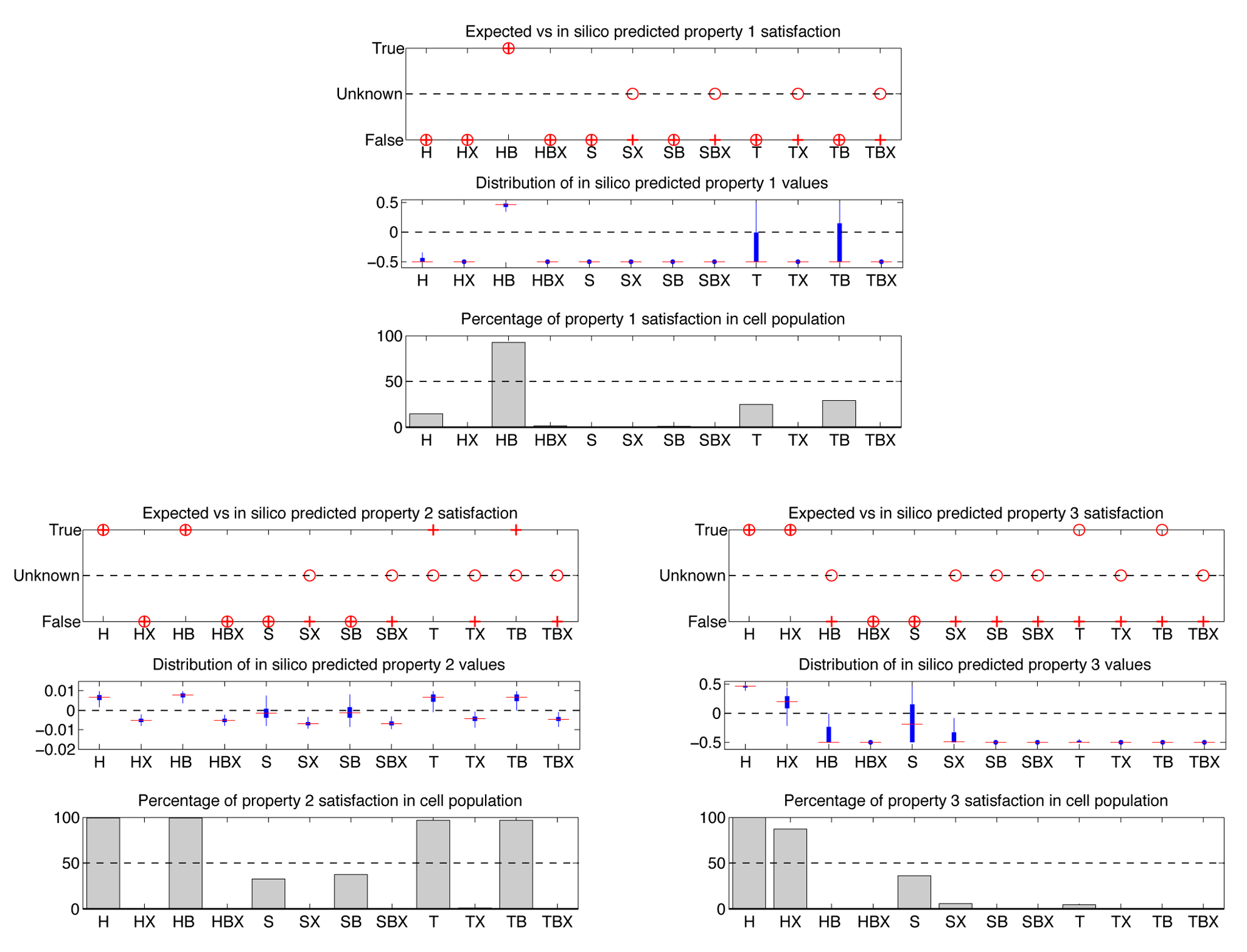

Supplement: Figure S5 — Population statistics for Property 1, 2 and 3, computed with new parameter values. (see Table S1) This data should be compared with Figure 4, 5 (right), and 6 (right). The new parameter values allow resolving the inconsistencies found for SKW6.4, OEBcl2 SKW6.4 cells for Property 2, and for ΔXIAP HCT116 cells for Property 3. T47D cells still do not satisfy Property 3 as expected. Notations are identical to those used in Figure 4. (TIF) [file pcbi.1003056.s005.tif]

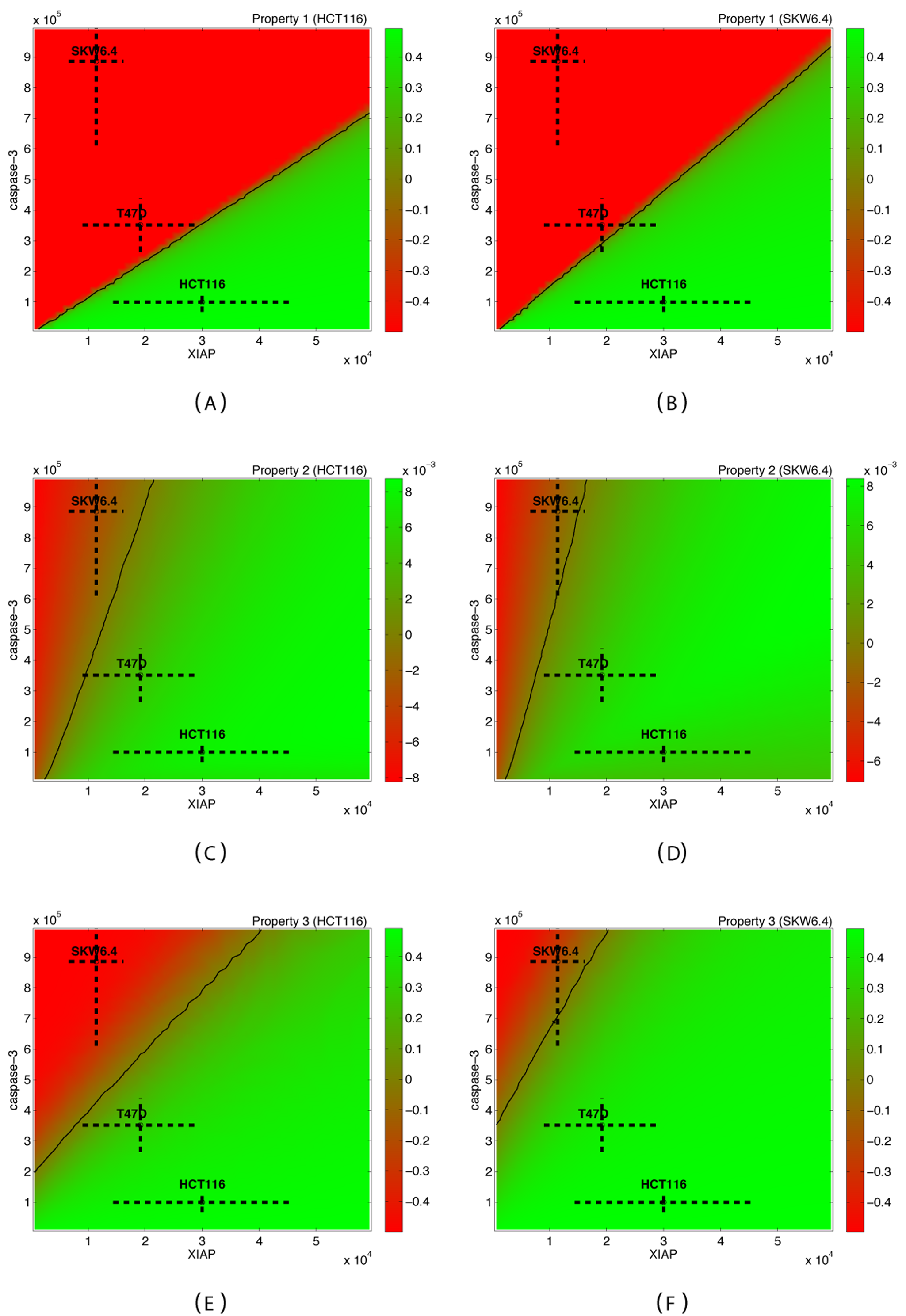

Supplement: Figure S6 — XIAP/Capsase-3 STL diagrams computed with new parameter values for all properties and using HCT116 or SKW6.4 as reference cell lines. Diagrams representing the values of the STL properties p1 (A–B), p2 (C–D) and p3 (E–F), computed using HCT116 (A,C,E) or SKW6.4 (B,D,F) nominal protein concentrations. (TIF) [file pcbi.1003056.s006.tif]

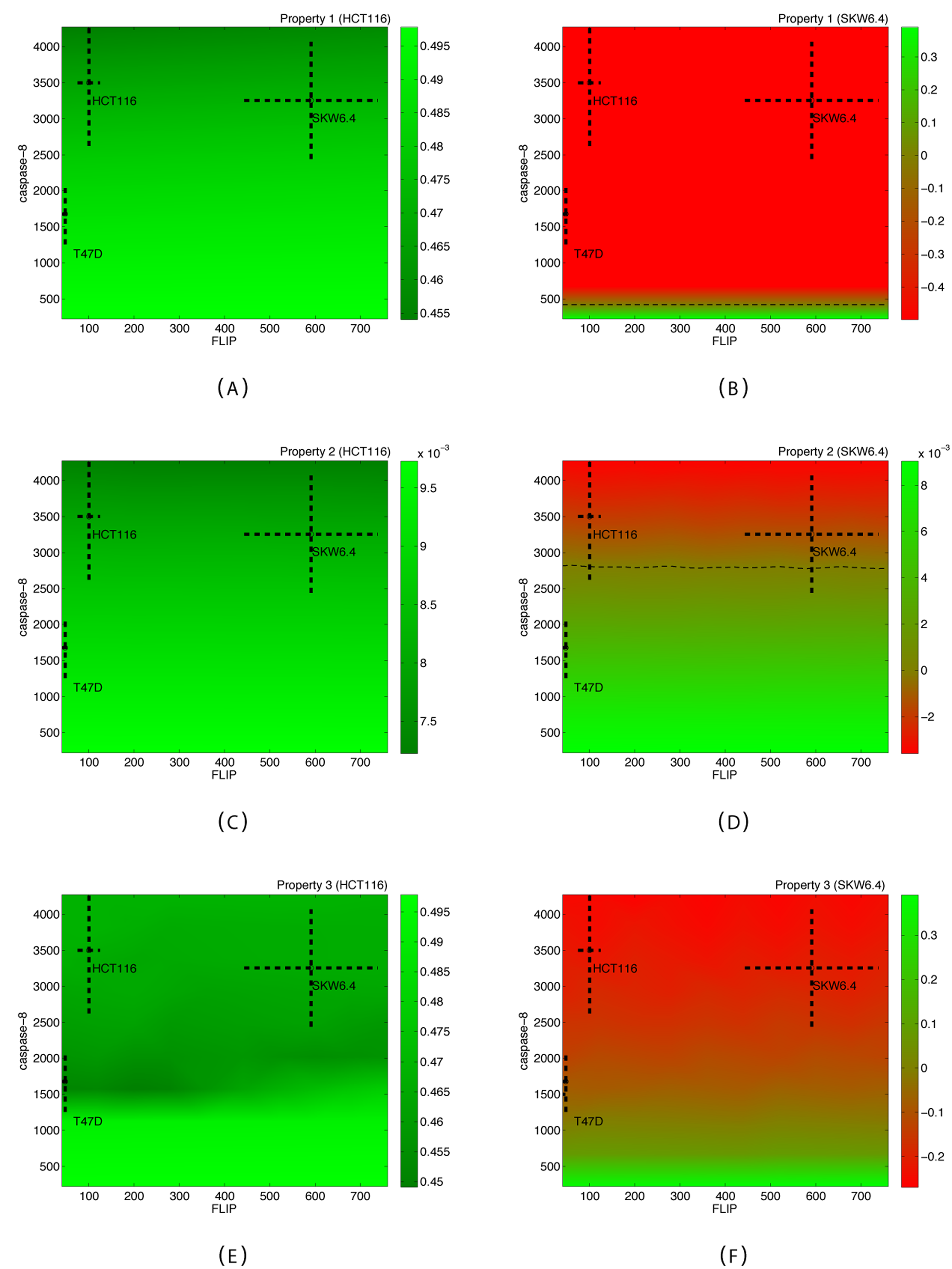

Supplement: Figure S7 — FLIP/Capsase-8 STL diagrams computed with new parameter values for all properties and using HCT116 or SKW6.4 as reference cell lines. Diagrams representing the values of the STL properties p1 (A–B), p2 (C–D) and p3 (E–F), computed using HCT116 (A,C,E) or SKW6.4 (B,D,F) nominal protein concentrations. (TIF) [file pcbi.1003056.s007.tif]

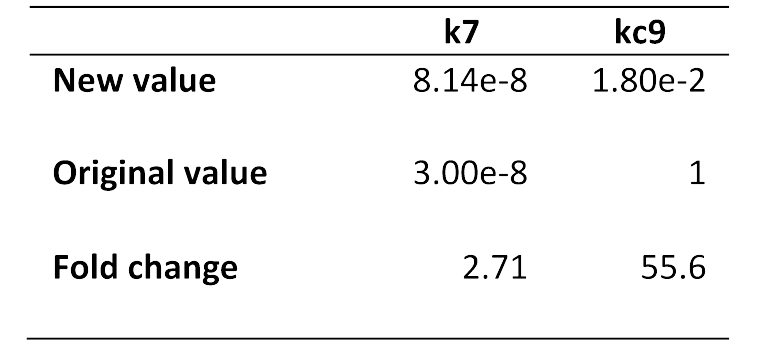

Supplement: Table S1 — Valid parameters. List of minimal parameter set leading to Property1–3 satisfaction for all but T47D cells, together with their new and original values, and the corresponding fold change. (TIF) [file pcbi.1003056.s009.tif]

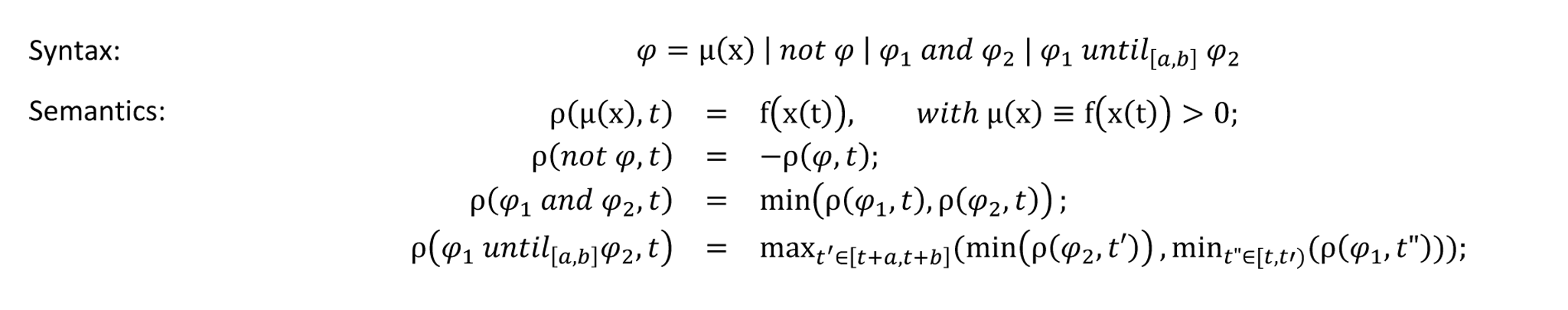

Supplement: Table S2 — Syntax and semantics of STL [48] . The syntax of STL formulas is defined inductively. Here, are STL formulas, is an equality of type , with f a real-valued function on the state x, and [a,b] is a time interval. The real-valued semantics of an STL formula φ at time t is interpreted on a real-valued signal x(t) defined on a time interval [0,Tf], where Tf is typically the end time of a simulation. One additionally defines as , as and as . (TIF) [file pcbi.1003056.s010.tif]
