# Supplementary material for: STL-based Analysis of TRAIL-induced Apoptosis Challenges the Notion of Type I/Type II Cell Line Classification
Source: PLoS Comput Biol. 2013 May 9;9(5):e1003056. doi: 10.1371/journal.pcbi.1003056 (PMC3649977; doi:10.1371/journal.pcbi.1003056)
Supplement: Program S1 — Computation of STL diagrams using Breach [33] . The archive contains the freely-distributed Matlab tool Breach, an implementation of EARM1.4 in Breach, initial conditions for each of 12 cell lines used in this article, and example scripts illustrating how to generate STL phase diagrams. (ZIP) [file pcbi.1003056.s008.zip › breach/Examples/iron/simplif3/data_simplif3.pdf]

Données sur les paramètres

Nicolas MOBILIA

21 juin 2012

Ce document présente les données que nous avons sur les paramètres et sur les concentration des variables à l'état stationnaire et en carence de fer pour le réseau simplif3.

Ce document se veut complètement exhaustif. En comparaison avec les données du système simplif1, les intervalles pour la concentration de TfR1 à l'état stationnaire et du taux de production basal de TfR1 ont été étendus.

La première section présente les données issues de données biologiques.

La deuxième section fournit des intervalles raisonnables sur les paramètres pour lesquels nous n'avons pas de données.

1 Données certaines

Les données (ou contraintes) issues du fichiers paramètres.pdf permettant de définir l'intervalle de valeur possible pour des variables et paramètres sont les suivantes :

cs1. $1,0.10^{-13} < k_{TfR1\_prod} < 2,0.10^{-13}$ mol/L/s

cs2. $k_{FPN1a\_deg} < 9,6.10^{-6}$ s^{-1}

cs3. $1,28.10^{-5} < k_{IRP\_deg} < 1,6.10^{-5}$ s^{-1}

cs4. $2,0.10^{-5} < k_{TfR1\_deg} < 3,0.10^{-5}$ s^{-1}

cs5. $Tf_{sat} = 0,3$ (à l'état stationnaire)

cs6. $4,2.10^{-5} < k_{IRP \to TfR1} < 14,4.10^{-5}$ s^{-1}

cs7. $2.10^{-2} < k_{Fe\_input} < 3,9.10^{-2}$ s^{-1}

cs8. $Fe \leq 2.10^{-6}$ mol/L

cs9. $3.10^{-9} < IRP < 10,7.10^{-9}$ mol/L (à l'état stationnaire)

cs10. $1,0.10^{-8} < TfR1 < 10,0.10^{-8}$ mol/L (à l'état stationnaire)

cs11. $k_{Fe\_cons} \geq k_{Fe\_export} \cdot FPN1a$ (à l'état stationnaire)

cs12. $n_{Ft} < 4500$

Si la borne minimum n'est pas précisée, celle-ci est fixée par défaut à 0.
Lorsque nous notons "(à l'état stationnaire)" cela signifie à l'état stationnaire correspond à la situation en présence de fer.

2 Données incertaines

Par analogie, nous proposons des intervalles de valeur acceptables pour les variables et paramètres suivants :

ci1. $1.0e-18 < k_{Ft\_prod} < 1.0e-10$ (1)

ci2. $1.0e-18 < k_{FPN1a\_prod} < 1.0e-10$ (1)

ci3. $1.0e-18 < k_{IRP\_prod} < 1.0e-10$ (1)

ci4. $1.0e-9 < k_{Ft\_deg} < 1.0e-2$ (2)

ci5. $0 < k_{Fe\_cons} < 1.0$ (3)

ci6. $0 < k_{Fe\_export} < 1.0e5$ (4)

ci7. $1.0e-9 < k_{Fe \to IRP} < 1.0$ (5)

ci8. $1.0e-13 < Ft < 1.0e-5$ (6)

ci9. $1.0e-13 < FPN1a < 1.0e-5$ (6)

ci10. $1.0e-13 < \theta_{IRP \to Ft} < 1.0e-5$ (7)

ci11. $1.0e-13 < \theta_{IRP \to FPN1a} < 1.0e-5$ (7)

ci12. $1.0e-13 < \theta_{fer \to IRP} < 1.0e-5$ (7)

ci13. $1.0e-19 < k_{IRP \to Ft} < 1.0e-10$ (8)

ci14. $1.0e-19 < k_{IRP \to FPN1a} < 1.0e-10$ (8)

Justifications :

– (1) : les valeurs de taux de production que nous avons sont de l'ordre de 0.1e-13 et 1.0e-14. Nous supposons qu'il n'y a pas de production plus de 1000 fois plus importante ou 1000 fois moins importante.
– (2) : les valeurs de taux de dégradation que nous avons sont de l'ordre de grandeur de 1.0e-5 et 1.0e-6.
– (3) : le terme positif de l'équation du fer et compris entre 3,24e-10 et 6,32e-10. Si nous considérons que la concentration en fer n'est pas inférieure à 1e-10, indiquer que le paramètre de consommation du fer compris en 0 et 1 est un encadrement correct.
– (4) : la sortie du fer via FPN1 est -a priori- faible. Nous mettons comme borne maximum 1.0e5, sans justification spécifique.
– (5) : en supposant que la concentration d'IRP2 étant de l'ordre de 1.0e-9 à 1.0e-8, considérer que $k_{fer \to IRP2}$ est compris entre 1.0e-9 et 1.0 est une bonne sur-approximation.
– (6) : les concentrations de protéines sont d'environ 1.0e-9. Nous prenons une grosse sur-approximation.
– (7) : la concentrations des IRP et du fer est compris dans cette plage. Les seuils sont de l'ordre des concentrations des protéines/atomes.
– (8) : Les IRPs empêchant la traduction des ARNs, la régulation maximale est au plus égale au taux de production. Il paraît raisonnable de considérer que la régulation n'est pas inférieur à 10 fois le taux de production (ce qui signifierait que la régulation par les IRP n'aurait que peut d'impact).

Nota, le paramètre n_{Ft} n'est pas utilisé pour le calcul de l'état stationnaire, mais intervient lors des simulations traduisant l'évolution du système en présence de fer.
